# An Eleven-Point Ultrasound-Guided Fascia Hydrorelease Protocol for Non-Odontogenic Toothache and Orofacial Pain: A Clinical Protocol

**DOI:** 10.3390/medicina62091808

**Published:** 2026-09-19

**Authors:** Hiroaki Kimura, Tadashi Kobayashi, Ryoya Asaka, Hideaki Obata

**Affiliations:** 1 Kimura Pain Clinic (Medical Corporation Fascia Research Association), Maebashi 371-0013, Gunma, Japan; pt.asaka63@gmail.com; 2 Development of Community Healthcare, Hirosaki University Graduate School of Medicine, Hirosaki 036-8562, Aomori, Japan; koba_mode_mail@yahoo.co.jp; 3 Department of Anesthesiology, Saitama Medical Center, Saitama Medical University, Kawagoe 350-8550, Saitama, Japan; hoobata@gmail.com

**Keywords:** non-odontogenic toothache, orofacial pain, ultrasound-guided fascia hydrorelease, Fascial Pain Syndrome, parotid fascia, cervical sympathetic ganglion, clinical protocol

## Abstract

*Background and Objectives:* Non-odontogenic toothache often persists without an identifiable dental cause and may lead to unnecessary pulpectomy or tooth extraction. It may also result in long-term combined use of psychotropic medications, forming a recurrent clinical pattern. Ultrasound-guided fascia hydrorelease (US-FHR) targeting fascia-derived pathology has been applied to non-odontogenic toothache; however, treatment regions have not been systematically described. This article presents a proposed eleven-point US-FHR protocol for non-odontogenic toothache and related orofacial pain. *Materials and Methods:* This is an experience-based clinical protocol; it is not an efficacy study and does not include a new prospective study on human participants. The protocol was organized on the basis of long-term clinical experience at Kimura Pain Clinic and a focused literature search. *Results:* The protocol comprises 11 POINTs distributed across six anatomical regions: (1) three POINTs in the masticatory muscle region, (2) three POINTs in the medial pterygoid and capsular region, (3) one POINT in the parotid region, (4) two POINTs in the cervical region, (5) one POINT in the facial-artery region, and (6) one POINT in the upper posterior cervical region. For each POINT, the anatomical rationale, referred-pain pattern, procedural concept, and safety considerations are described. *Conclusions:* The proposed eleven-point protocol represents an expanded and structured development of the earlier four-region approach. Prospective, ideally controlled, studies are needed to evaluate its clinical effectiveness.

## 1. Introduction

Non-odontogenic toothache is defined as pain perceived in the dental region but originating from non-dental tissues. The Japanese Society of Orofacial Pain’s clinical guideline [1] categorizes it into eight types: (i) muscular/myofascial toothache, (ii) neuropathic toothache, (iii) neurovascular toothache, (iv) maxillary sinus-related toothache, (v) cardiac toothache, (vi) toothache associated with psychiatric disorders or psychosocial factors, (vii) idiopathic toothache, and (viii) toothache caused by various other diseases. Among these, category (i) reflects pain of myofascial origin; however, this definition does not address fascia-derived pathology in the broader sense, which encompasses aponeuroses, paraneural sheath and fascia around the nerve, perivascular fascia, spaces between fasciae, and fat pads [2].

In our 2020 Dental Diamond article [3], we proposed that fascial abnormalities—including stacking, densification, and adhesion—can act as primary pain generators in non-odontogenic toothache. We described the procedures of ultrasound-guided fascia hydrorelease (US-FHR) in four representative treatment regions (Kimura et al., 2020) [3]: (1) temporalis/lateral pterygoid/masseter, (2) medial pterygoid/fat pad, (3) temporomandibular joint capsule complex, and (4) sternocleidomastoid/levator scapulae. The present article builds upon this foundation, drawing on subsequent clinical experience at the corresponding author’s institution, in which these four regions have been progressively reorganized into a proposed eleven-point protocol (including POINT 11, which addresses vascular and perivascular structures); the rationale for each intervention point is systematically presented from the perspective that fascial pathological changes (densification, stacking, and impaired gliding) are clinically common contributors to such pain.

Newly described POINTs include an intraoral approach to the medial pterygoid, release of the superficial parotid fascia and an integrated approach to the cervical sympathetic ganglion region, the upper posterior cervical region, the digastric muscle, the pterygopalatine fossa, and the perivascular fascia of the facial artery.

The recognition that fascia—a continuous connective tissue system encompassing the muscular fascia, aponeuroses, paraneural sheath and fascia around the nerve, perivascular fascia, and other related structures [2]—can serve as a possible source of pain and has not been sufficiently addressed in contemporary medical and dental education. As a result, patients with non-odontogenic toothache that has become chronic and labeled as “pain of unknown origin” frequently fall into the following recurrent clinical pattern:

(i) Repeated unnecessary pulpectomy and tooth extraction after the exclusion of odontogenic origins;
(ii) Transition to pharmacological management labeled as psychogenic or neuropathic pain;
(iii) Long-term combined use of antidepressants, anxiolytics, and hypnotics;
(iv) Symptom complexity arising from psychotropic medications and polypharmacy;
(v) Persistent oversight of fascia as a possible source of pain, leading to chronification.

The systematization presented in this article responds to this clinical reality by highlighting fascia as a potential contributing structure of pain and by providing an anatomical and procedural framework as a shared interdisciplinary language for pain physicians, dentists, physical therapists, and acupuncturists.

Furthermore, in a previous cadaveric study (10 cadavers, 20 injections), the corresponding author and colleagues demonstrated that 1.0 mL of dye solution injected under ultrasound guidance spreads over a wide area between epimysiums, measured as 24.50 cm^2^ and 18.82 cm^2^ on the deep and superficial sides, respectively [4]. This finding provides the biomechanical rationale for the small injection volume of 1–2 mL used at each POINT in the present protocol. The same study also identified the out-of-plane (oblique) approach as the most accurate technique for injection between epimysiums, supported by anatomical verification.

A recent anatomical study by Shiwaku, Pirri, Stecco and colleagues [5] provides further support. Using five fresh-frozen cadavers and eight limbs, they demonstrated that 2.5 mL of saline injected under ultrasound guidance mainly distributes into the space between the aponeurotic fascia (APF) and epimysium (EPI). This finding offers an important anatomical foundation for the APF–epimysial targeting strategy adopted in the present protocol. This article integrates these recent findings and organizes them as an eleven-point ultrasound-guided fascia hydrorelease protocol for non-odontogenic toothache and orofacial pain.

### Aim of the Study

The aim of the present article is to present the currently proposed eleven-point protocol, developed on the basis of a focused review of the specialty literature and of accumulated clinical practice, and to provide an anatomical and procedural framework as a shared language for pain physicians, dentists, physical therapists, and acupuncturists. This is an experience-based clinical protocol, not an efficacy study. Performance of the injection procedures themselves is restricted to physicians with adequate training in ultrasound-guided interventions, in accordance with the legal regulations and professional scopes of practice of each country. Within this framework, dentists play a central role in the diagnosis, exclusion, and referral of non-odontogenic toothache, and physical therapists and acupuncturists collaborate in assessment and post-procedure rehabilitation.

## 2. Background

### 2.1. Definition of Fascia

Anatomically speaking, fascia is a comprehensive concept (Stecco and Schleip [2]) encompassing myofascia, retinacula, ligaments, tendon sheaths, joint capsules, fat pads, peripheral neural fascia, meningeal fascia, the dura mater–ligamentum flavum complex, and vascular fascia. The Stecco group has proposed the concept of the fasciatome—analogous to the cutaneous dermatome—to organize the functional fascial territories supplied by each nerve root [6].

### 2.2. Fascial Pain Syndrome (FPS)

Pain of myofascial origin has traditionally been described as myofascial pain syndrome (MPS) (Travell, Simons & Simons’, 3rd ed. [7]). However, pain generators are increasingly recognized in fascial structures other than myofascia. In July 2019, we proposed Fascial Pain Syndrome (FPS) as a successor concept to MPS [8]. FPS is characterized by sensory, motor, and autonomic symptoms caused by fascial abnormalities, and its treatment requires identification of the fascia acting as the pain generator. On ultrasound, the putative pain-generating fascia is observed as a white, thickened, hyperechoic image referred to as stacking fascia [3].

### 2.3. Ultrasound-Guided Fascia Hydrorelease (US-FHR)

US-FHR is a procedure in which an abnormal fascia is identified under ultrasound, released with an injection of saline or some other solution, providing analgesia together with improved fascial flexibility (extensibility and gliding). It is a representative treatment technique for FPS [3]. The term fascia hydrorelease is of Japanese origin and is not yet an established international standard term; it is retained here, together with US-FHR, as the defined name of the technique. According to fascial adhesion strength, a graded therapeutic hierarchy from Grade 0 (very weak, manual) to Grade 4 (very strong, surgery) has been proposed. US-FHR occupies the Grade 2–3 range and consists of a precise injection of saline or bicarbonate Ringer’s solution through a 27–30 G fine needle.

### 2.4. Anatomical Rationale for Fascia-Derived Non-Odontogenic Toothache

The masticatory muscles (masseter, temporalis, lateral pterygoid, and medial pterygoid) are innervated by the third division of the trigeminal nerve (mandibular nerve) and are classically known to elicit strong referred pain to the dental region [7]. Wright (2000) [9] quantitatively documented, in 230 patients with temporomandibular disorder (TMD), referred-pain patterns from masticatory muscle trigger points (TrPs) projecting to broad orofacial regions including the teeth. In women with TMD, Fernández-de-las-Peñas et al. (2010) [10] showed that referred-pain areas from TrPs in the masticatory and neck–shoulder muscles were significantly enlarged compared with controls. Svensson and Graven-Nielsen (2001) [11] comprehensively reviewed the mechanisms of craniofacial muscle pain and its referred pain in dental and facial regions. In the cadaveric anatomical study approved by the Nihon University Ethics Committee (approval number 28-8-0), it was shown that the temporalis attaches not only to the tip of the coronoid process but also broadly to the anterior and posterior surfaces of the coronoid process and the lateral pterygoid plate of the sphenoid, wrapping around the mandible; the lateral pterygoid attaches to the surface and posterior margin of the lateral pterygoid plate; the medial pterygoid attaches to the anterior and posterior surfaces of the lateral pterygoid plate and to the maxilla; and fat pads are present between these muscle layers [3]. The broad insertion sites of these muscles and the fat pads between them constitute an anatomical environment in which fascial densification and stacking are prone to develop, providing the anatomical basis by which these putative TrP/fascial pain sources generate referred pain from the masticatory region to the dental region—that is, fascia-derived non-odontogenic toothache.

The present protocol clinically targets—under ultrasound guidance—the fascial pathologies (such as densification between epimysiums and fat-pad adhesion) that are considered to underlie the referred-pain patterns classically described as TrP-related.

### 2.5. Anatomical Predilection Sites of Stacking Fascia (Memory Reset Hypothesis)

In our prior paper outlining the Fascial Memory Reset Hypothesis [12], stacking fascia—the macroscopic layered structural phenotype visualized on ultrasound—was interpreted as the mechano-epigenetic structural memory formed under sustained mechanical stress. In Section 3.1 of that paper [12], eight anatomical predilection sites for stacking fascia were summarized as follows:

• #1 Curved regions of tissues—e.g., curvature of the vertebral artery, facial artery bending, and curvature of the masseter muscle fibers.
• #2 Crossing points of tissues—e.g., crossing of the masticatory and hyoid muscles.
• #3 Convergence zones of multiple tissues—e.g., temporomandibular joint capsule complex (convergence of capsule, ligaments, and muscle attachments) and temporalis insertion.
• #4 Peritubular regions of nerves and vessels—e.g., pterygopalatine fossa (maxillary nerve and artery passage) and carotid sheath.
• #5 Periarticular fat pads—e.g., fat pad between the masseter and lateral pterygoid (buccal fat pad).
• #6 Superficial course of neurovascular structures—e.g., superficial parotid region (the facial nerve and parotid duct run beneath the superficial fascia [the superficial musculoaponeurotic system, SMAS]) and superficial course of the facial artery.
• #7 Ligamentum flavum and epidural space—e.g., C0–1 posterior atlanto-occipital membrane and C1–C2 ligamentum flavum/dural region.
• #8 Predilection sites for accessory muscles—e.g., accessory masticatory muscles.

Each treatment point in the present eleven-point protocol corresponds to one or more of these eight categories (often a combination) and is thereby anatomically grounded. In particular, integrated POINT 3 (lateral pterygoid/fat pad/maxillary artery/pterygopalatine fossa) corresponds to the composite of #1 + #4 + #5 + #6, POINT 11 (upper posterior cervical) corresponds to #7 (PAOM and ligamentum flavum) + #1 (vertebral artery curvature) + #6 (perivascular fascia of the vertebral artery), and POINT 7 (superficial parotid) corresponds to #6 (superficial course of neurovascular structures). The corresponding category is explicitly indicated at each POINT in Section 6.

## 3. Methods: Protocol Development Framework

In terms of study design, this is an experience-based clinical protocol article that does not include a new prospective study on human participants; it is not an efficacy study. This article synthesizes the eleven-point protocol from two complementary sources: (i) a focused literature search of the fascia, orofacial pain, and ultrasound-guided fascia hydrorelease domains, and (ii) the corresponding author’s over-a-decade clinical experience encompassing an estimated 360,000 or more US-FHR procedures across all anatomical regions, of which a substantial portion involved orofacial and cervical structures relevant to non-odontogenic toothache. Rather than a systematic review, this work presents a clinically grounded synthesis aimed at articulating an actionable intervention framework.

The literature search was conducted in PubMed and Ichushi Web from January 2010 to May 2026 (final search: May 2026; English- and Japanese-language publications), using the keywords “non-odontogenic toothache,” “fascia,” “ultrasound-guided injection,” “masticatory muscle pain,” “trigger point,” and related variants. Articles were prioritized when they (a) described relevant fascial anatomy or pathophysiology, (b) reported clinical outcomes of fascial or myofascial interventions, or (c) provided foundational anatomical or histological evidence underpinning the proposed intervention points. Both peer-reviewed publications and authoritative monographs (Travell-Simons, Stecco group, JNOS publications) were included. Authoritative monographs and society publications were selected according to the following principle: standard anatomical textbooks and the official publications of the relevant professional societies were used, restricted to statements of descriptive anatomy and officially adopted definitions. No further formal selection protocol was applied.

The clinical experience component was derived from the corresponding author’s clinical practice at a specialized pain clinic (Kimura Pain Clinic, Maebashi, Japan), encompassing over a decade of practice and an estimated 360,000 or more US-FHR procedures across all anatomical regions (a rough estimate of procedural workload: approximately 150 procedures/day × 20 days/month × 12 months × 10 years; not derived from a verified procedural registry, and not representing numbers of patients, treatment sessions, or orofacial procedures). Procedures were performed under real-time ultrasound guidance. The eleven-point selection was progressively refined through this accumulated practice, evolving from the previously published four-region approach (Kimura et al., 2020) [3] by adding targets that recurred in the corresponding author’s clinical practice for non-odontogenic toothache; each added target was retained only when the safety-margin criteria described below were met, resulting in the present eleven POINTs, with POINTs 3, 5, 7, 9, 10 and 11 as new or substantially expanded targets.

Specific criteria for inclusion in the final eleven-point set were: (a) anatomical correspondence to one or more of the eight stacking-fascia categories described in Section 2.5; (b) reproducible identification of densified (stacking-pattern) fascia at the proposed site on high-frequency ultrasound; (c) consistent clinical responsiveness to US-FHR observed across the corresponding author’s accumulated case experience; and (d) sufficient safety margin given the regional neurovascular anatomy. For criterion (d), a candidate point was regarded as having a sufficient safety margin when (i) the relevant major vessels and nerves are identifiable by ultrasound (color Doppler) at that site, (ii) an approach path allowing continuous needle-tip visualization away from those structures exists, and (iii) the target interfascial plane can be reached at a depth at which tip visibility is maintained with the needles used. Sites that did not meet these criteria, or for which clinical responsiveness was inconsistent, were not included in the final protocol.

This synthesis is presented as a clinically grounded clinical protocol article rather than a systematic review. The methodology aims to articulate an actionable framework based on accumulated clinical practice, and does not include formal grading of evidence quality or quantitative synthesis. Instead, the basis of each statement is distinguished descriptively—as established (direct) evidence, anatomical rationale (indirect evidence), expert clinical experience, or biological hypothesis—as set out in Section 8.1, so that the level of support can be assessed for each POINT individually. The protocol presentation itself (Section 6 and Section 7) constitutes the deliverable of this article.

## 4. Diagnosis and Evaluation

This section briefly summarizes the diagnostic reasoning required to provide clinical context for the subsequent treatment protocol. A comprehensive review of differential diagnosis is beyond the scope of this article.

### 4.1. Clinical Presentation

**Figure 1:**
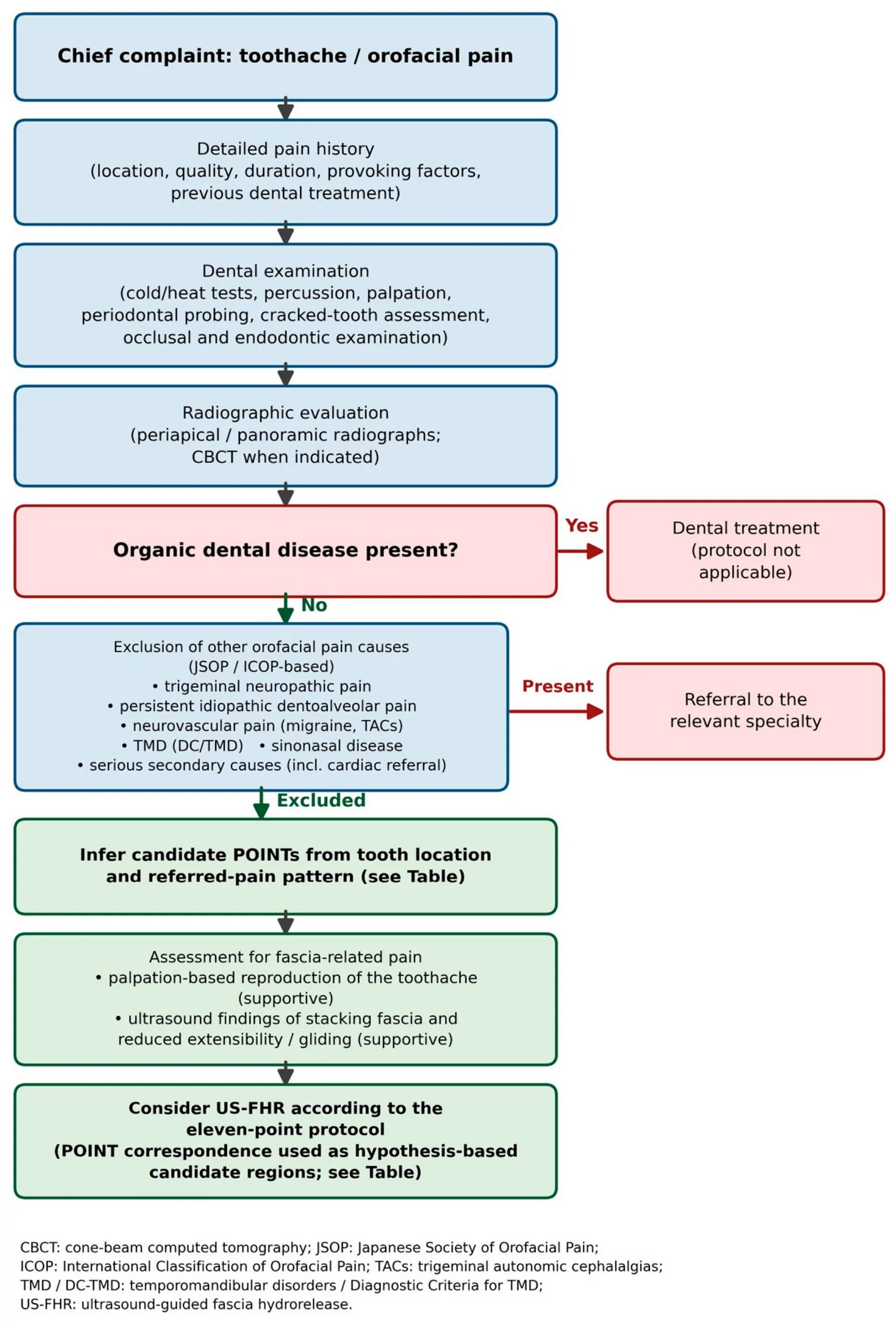
Diagnostic algorithm for non-odontogenic toothache: stepwise exclusion of odontogenic and non-fascial causes before consideration of ultrasound-guided fascia hydrorelease. The algorithm incorporates the International Classification of Orofacial Pain (ICOP) [13] and the Diagnostic Criteria for Temporomandibular Disorders (DC/TMD) [14]. Neither the palpation-based reproduction of the toothache nor the ultrasound findings of stacking fascia is confirmatory; sensitivity and specificity have not been established.

Non-odontogenic toothache is clinically diagnosed as persistent dental region pain that remains after dental examinations (pulp electric testing, occlusal examination, and imaging) have excluded organic dental disease. Within the diagnostic framework of the Japanese Society of Orofacial Pain (2019) [1], the authors propose that fascia-derived pain sources be considered as a candidate cause, especially in cases of refractory pain that is not improved by conventional dental treatment. In addition to the classification of the Japanese Society of Orofacial Pain [1], the International Classification of Orofacial Pain (ICOP) [13] and the Diagnostic Criteria for Temporomandibular Disorders (DC/TMD) [14] provide internationally standardized frameworks for the differential diagnosis of orofacial pain—including trigeminal neuropathic pain, persistent idiopathic dentoalveolar pain, and neurovascular orofacial pain—and a systematic review by Yatani et al. [15] provides recommendations for the diagnostic pathway of non-odontogenic toothache; the diagnostic reasoning summarized in this section is consistent with these frameworks (Figure 1).

Clinical features include (a) an absence of dental abnormality on dental examination, (b) typical referred-pain patterns (masseter → maxillary/mandibular molars; temporalis → maxillary molars and temple; medial pterygoid → mandibular molars and tongue; perivascular facial artery → mandibular anterior region), (c) trigger-point-like tenderness on palpation of the masticatory, parotid, cervical, or perivascular regions, (d) visualization of stacking fascia on ultrasound, and (e), when elicitable, reproduction of the patient’s dental pain by palpation of the candidate pain source is supportive (sensitivity and specificity of these findings for fascia-related pain have not been established).

### 4.2. Pain Sources Classified by Tooth Location and Referred Pattern

Based on the location of the dental pain and the referred-pain pattern, candidate pain-source muscles and fasciae can be inferred. Classically, Travell, Simons and Simons [7] systematized the dental referred-pain patterns of masticatory muscle trigger points (TrPs). In 230 patients with temporomandibular disorder (TMD), Wright (2000, J Am Dent Assoc) [9] quantitatively documented referred-pain patterns from masticatory muscle TrPs projecting to broad orofacial regions including the teeth. Fernández-de-las-Peñas et al. (2010, J Pain) [10] showed quantitatively, in women with TMD, that the referred-pain areas of TrPs in the masticatory and neck–shoulder muscles are significantly enlarged compared with controls. Svensson and Graven-Nielsen (2001, J Orofac Pain) [11] comprehensively reviewed the mechanisms of masticatory muscle pain and its referred-pain projection to dental and facial regions. Under ultrasound guidance, the present protocol clinically targets the fascial pathologies (such as densification between epimysiums and fat-pad adhesion) that are considered to underlie these referred-pain patterns. The following mapping presents hypothesis-based candidate regions, not a validated clinical decision rule.

• Maxillary molar region: Masseter (POINT 1), temporalis (POINT 2), medial pterygoid (POINTs 4–5), superficial parotid (POINT 7), and lateral pterygoid/pterygopalatine (POINT 3 integrated POINT).
• Maxillary premolar region: Masseter (POINT 1), medial pterygoid (POINTs 4–5), superficial parotid (POINT 7), and lateral pterygoid/pterygopalatine (POINT 3).
• Maxillary anterior teeth: Superficial parotid (POINT 7) and lateral pterygoid/pterygopalatine (POINT 3).
• Mandibular molar region: Masseter (POINT 1), medial pterygoid (POINTs 4–5), TMJ capsule (POINT 6), and perivascular fascia of the facial artery (POINT 10).
• Mandibular premolar region: Medial pterygoid (POINTs 4–5), digastric (POINT 8), and perivascular fascia of the facial artery (POINT 10).
• Mandibular anterior teeth: Digastric (POINT 8) and perivascular fascia of the facial artery (POINT 10).
• Toothache associated with periarticular symptoms: Lateral pterygoid (POINT 3), TMJ capsule (POINT 6), and medial pterygoid (POINTs 4–5).
• Refractory cases with referred pain from upper teeth to deep eye to temple: Upper posterior cervical (POINT 11, Advanced).
• Toothache with swallowing pain or lingual discomfort: Digastric (POINT 8).
• Toothache with headache or autonomic symptoms: Cervical sympathetic ganglion region (POINT 9).

## 5. Ultrasound-Guided Fascia Hydrorelease (US-FHR) Procedure

### 5.1. Principles

In the present protocol, ultrasound-guided fascia hydrorelease (US-FHR) is performed using a linear probe (or, depending on the site, in combination with a convex probe) to identify suspected pain-generating (stacking) fascia and to inject 1–2 mL of physiological saline or bicarbonate Ringer’s solution into the space between epimysiums using a 27 G 38 mm needle (30 G is used at POINTs 3, 5, 7, 10, and 11). Based on anatomical evidence of its superior accuracy, the out-of-plane (oblique) approach is used as standard [4]. Bicarbonate Ringer’s solution may be selected to reduce injection-related pain owing to its buffering effect; the choice between physiological saline and bicarbonate Ringer’s solution is otherwise based on availability and cost.

### 5.2. Safety Considerations

In the present protocol, safety is supported by (1) the use of fine 27–30 G needles via the out-of-plane technique (30 G at POINTs 3, 5, 7, 10, and 11), (2) color Doppler identification of vascular structures prior to each release (Doppler is then turned off to optimize B-mode needle visualization), (3) continuous visualization of the needle tip under ultrasound guidance, (4) careful avoidance of intravascular and intraneural injection, and (5) immediate cessation of injection if abnormal resistance is felt. POINT-specific cautions are described in the respective sections. With the out-of-plane approach, the needle tip is distinguished from the needle shaft and identified by three concrete techniques: probe tilt/toggle to bring the tip echo in and out of the beam, hydrolocation with a minimal test injection, and stepwise advancement with the tip kept within the beam. In addition, an explicit stopping rule is applied: the needle is not advanced unless the needle tip is clearly identified, and if identification of the needle tip cannot be regained, the procedure is terminated. Even under ultrasound guidance, complications such as transient neural injury and inadvertent intravascular injection have been reported during head and neck procedures [16]; continuous needle-tip identification and this stopping rule are therefore strictly applied. Risk stratification, contraindications, and complication management are summarized in Section 5.4 and Section 5.5 (Table 1 and Table 2). The precautions described here reduce, but do not eliminate, risk and do not constitute safety validation, which requires prospective study.

The standard descriptive format for each POINT is as follows [3]: (1) patient positioning, (2) the injection site indicated by a yellow arrow, and (3) the release area indicated by a yellow dashed line.

### 5.3. Anatomical Rationale for Needle Technique and Injection Volume [4]

All injections were performed using a 27 G needle via the out-of-plane (oblique) approach (30 G is used at POINTs 3, 5, 7, 10, and 11). The rationale for this selection is supported by the corresponding author’s previous cadaveric study (Kimura H et al., Pain Med 2020 [4]). The out-of-plane approach has the following advantages over the in-plane equivalent:

• (1) Allows the highest accuracy of injection into the space between epimysiums using a fine 27 G 38 mm needle.
• (2) The injection and dispersion origin are close to the puncture site, facilitating anatomical landmark identification during the procedure.
• (3) Avoids the upward spread along the needle path and the distribution accuracy loss that may occur with the in-plane approach.
• (4) Less invasive in clinical practice than the in-plane approach.
• (5) Modern ultrasound devices with simple needle visualization functions provide sufficient needle visibility even with the out-of-plane approach.

Regarding injection volume, the same cadaveric study demonstrated that only 1.0 mL of solution spreads across a wide area between epimysiums (median 24.50 cm^2^ on the deep side and 18.82 cm^2^ on the superficial side; *p* = 0.033). This provides the biomechanical rationale for the 1–2 mL volume used at each POINT in the present protocol.

### 5.4. Risk Stratification and Operator Requirements

The eleven POINTs are stratified into three levels—Basic, Intermediate, and Advanced—according to the regional neurovascular anatomy and the technical difficulty of maintaining continuous needle-tip visualization (Table 1). Performance of all injection procedures remains restricted to physicians with adequate training in ultrasound-guided interventions (Section 1).

### 5.5. Contraindications, Complications, and Their Prevention, Recognition, and Management

As listed in our previous article [3], general complications related to this procedure include vascular puncture with bleeding/hematoma, infection at the puncture site, post-injection pain, and delayed muscle soreness. Color Doppler identification of vessels, strict aseptic technique, the use of fine needles via the out-of-plane approach, and minimization of injection volume contribute to risk mitigation. Table 2 summarizes contraindications and common complications together with their prevention, recognition, and management; the per-POINT cautions in Section 6 supplement this table.

### 5.6. Technical Parameters

All procedures are performed with a Konica Minolta SONIMAGE HS ultrasound system (linear L18-4, 4–18 MHz; convex C5-2, 2–5 MHz). Common B-mode display settings, as shown in the on-screen parameter headers of the representative ultrasound images, are: frame rate FR 20–40, persistence P 100, tissue harmonic imaging THI On, high-resolution mode HRes, B-mode gain BG 20–40, and dynamic range DR 60–75; the imaging depth is site-dependent, as indicated by the depth scales of the figures. The standard approach is out-of-plane with continuous needle-tip visualization; the needle is not advanced unless the needle tip is clearly identified, and the procedure is terminated if identification cannot be regained (Section 5.2). With this out-of-plane approach, the skin entry point is placed adjacent to the midpoint of the probe, and the needle angle is adjusted according to the depth of the target fascial plane. Before each release, vascular structures are identified with color Doppler, after which Doppler is turned off to optimize B-mode needle visualization (Section 5.2). Prior to injection, aspiration and a minimal test injection (hydrolocation) are used to confirm the extravascular position of the needle tip. The injectate is physiological saline or bicarbonate Ringer’s solution; no local anesthetics are used. A maximum of four POINTs (usually three to four) are treated per session, with a total injectate volume of up to approximately 8 mL (four POINTs × 1–2 mL). Sessions are usually performed twice weekly (two to three times weekly when pain is severe), tapered according to improvement, and individualized. Patients are observed for 30–60 min after the procedure. The procedural end point is sonographic separation of the interfascial plane by the injectate, accompanied by reduction in tenderness and improvement of fascial mobility (Section 2.3); termination criteria are worsening pain, abnormal resistance, suspected vascular puncture, and loss of needle-tip visualization (Table 2). Table 3 summarizes the per-POINT parameters. Typical target depths are approximate values read from the depth scales of the representative figures.

## 6. The Eleven-Point Protocol

This chapter describes the eleven-point proposed protocol by region. Each POINT is described according to the following structure: anatomy, referred-pain pattern, ultrasound technique, and US-FHR procedure. Table 4 summarizes the supporting evidence for each POINT. Demonstration videos of the procedure at each POINT are provided as Supplementary Videos S1-S11 (POINT 11 as Videos S11A and S11B).

All eleven POINTs were selected on the basis of anatomical rationale and the corresponding author’s clinical experience; the anatomical rationale for each POINT is described in Section 6. In all POINTs, the identification of stacking fascia as the pain-generating structure is part of the published hypothesis framework [12] and remains to be validated prospectively. “Hypothesis-generating” indicates targets first proposed by the authors without published clinical reports of referred pain to the teeth.

### 6.1. Masticatory Muscle Region (POINTs 1–3)

The masticatory muscle region is a further development of POINT 1 from our previous article [3] (temporalis/lateral pterygoid/masseter). Through accumulated clinical experience, the masseter, temporalis, and lateral pterygoid have been recognized as independent pain sources and are described here as three separate POINTs.

POINT 1: Masseter (superficial and deep layers)

The masseter consists of a superficial and a deep layer, separated by a thin fascia between the two epimysial layers. Referred pain involves the upper and lower molars, preauricular region, and cheek [7]. The probe is held perpendicular to the inferior border of the zygomatic arch to image the masseter in the short axis.

**Figure 2:**
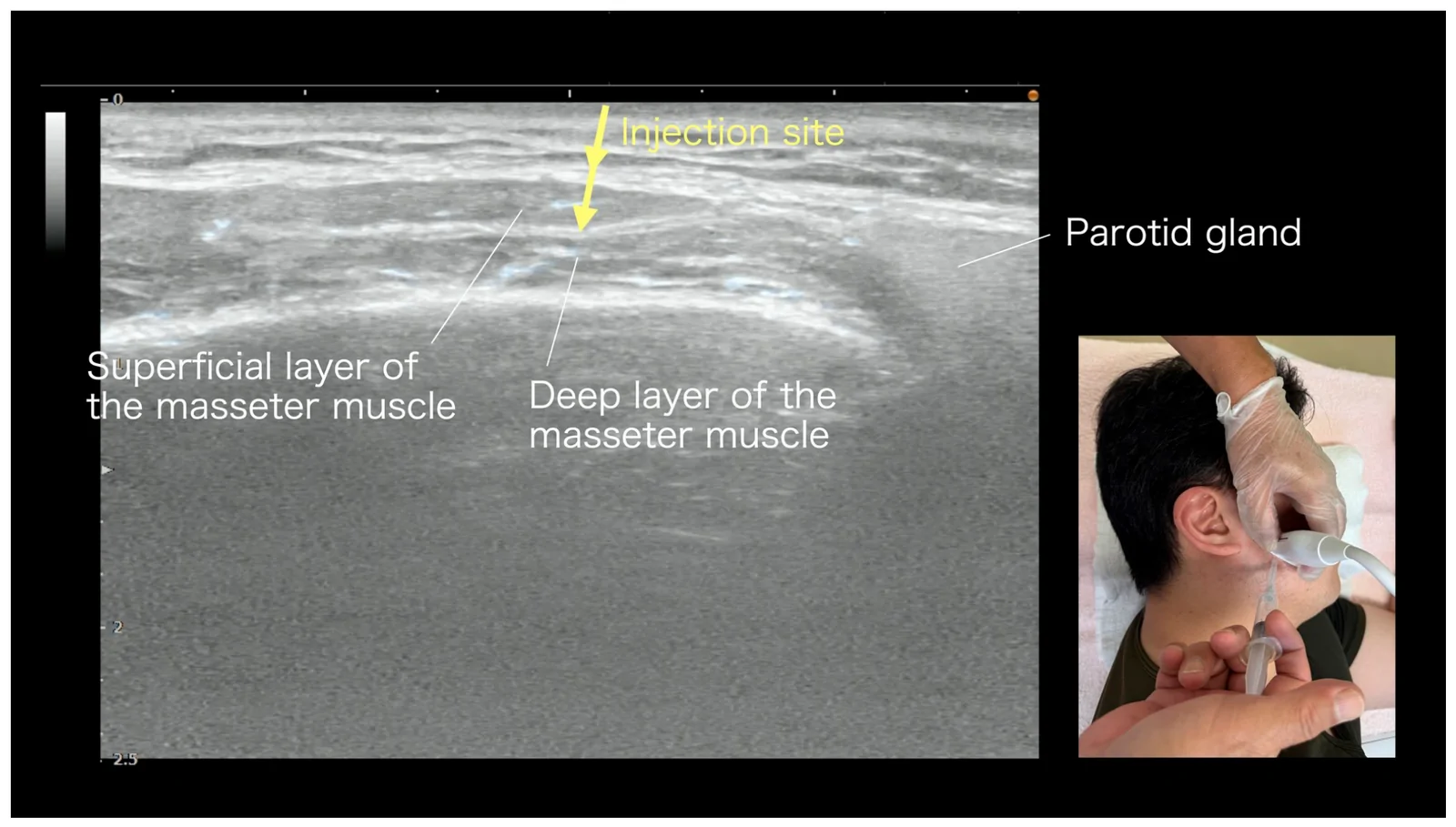
Masseter US-FHR (POINT 1). Ultrasound image: Superficial and deep layers of the masseter and the mandibular ramus are identified. The yellow arrow (injection site) indicates the plane between the superficial and deep epimysiums of the masseter. With a 27 G 38 mm needle, 1–2 mL of saline is injected via the out-of-plane approach. Color Doppler is used to confirm vascular structures before injection. The inset (bottom right) shows the patient position and probe placement.

• Anatomy: From the zygomatic arch to the lateral surface of the mandibular ramus; innervated by the third division of the trigeminal nerve.
• Referred pain: Upper and lower molars, preauricular region, cheek, and temple.
• Ultrasound: Inferior border of the zygomatic arch; a convex probe is advantageous for an overview.
• US-FHR: 27–30 G needle, 1–2 mL into the space between the superficial and deep epimysiums of the masseter; the target is superficial and distant from major neurovascular structures (Figure 2).

POINT 2: Temporalis (anterior and posterior, integrated)

The temporalis is a broad fan-shaped muscle with anterior, middle, and posterior functional subdivisions. Referred pain involves the maxillary molars, the temple, and the frontal region, and often presents as tension-type-headache-like symptoms [7]. The probe is positioned over the temporal region above the zygomatic arch.

**Figure 3:**
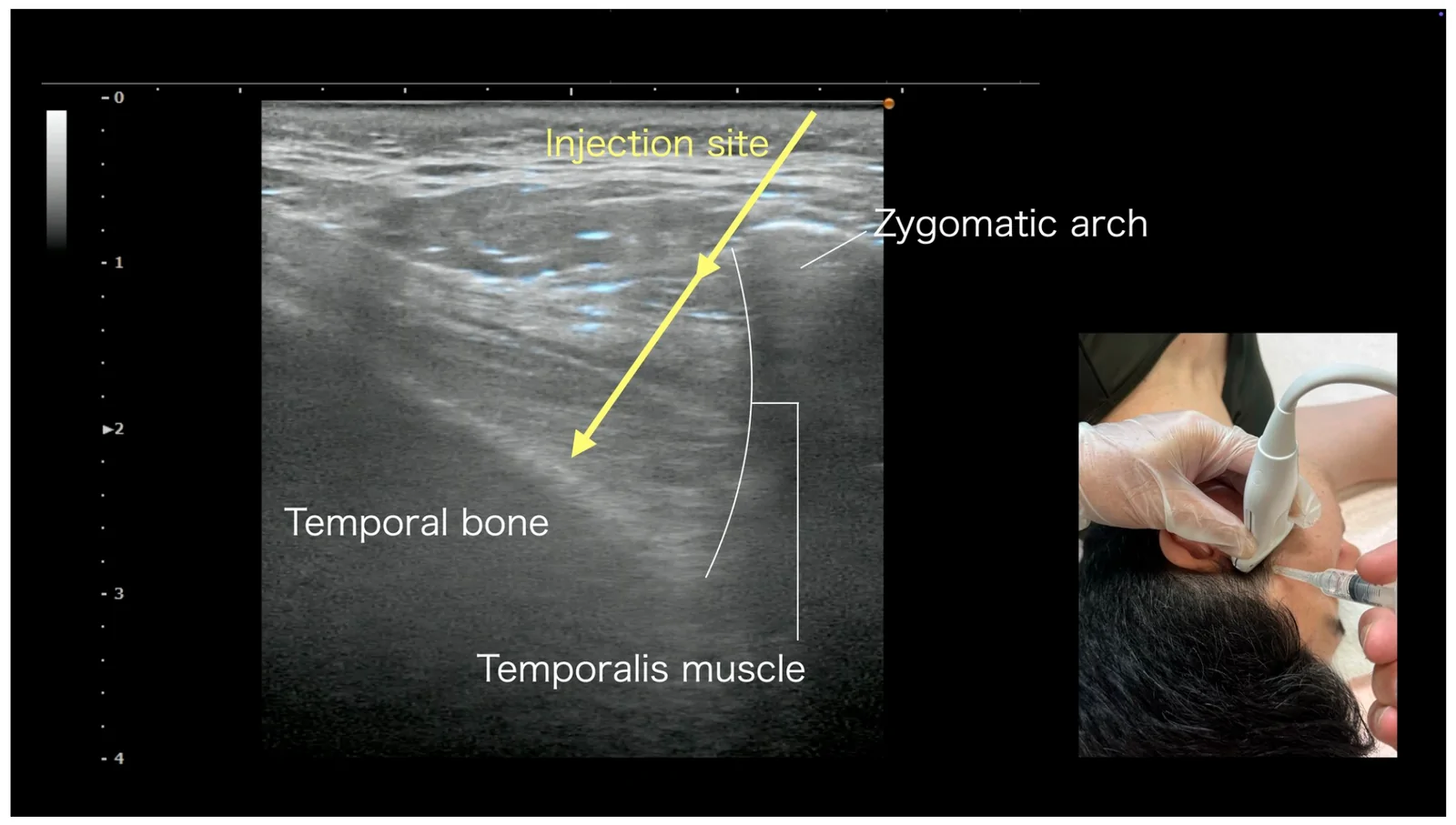
Temporalis US-FHR (POINT 2). Ultrasound image: Temporalis muscle, zygomatic arch, and temporal bone are identified. The yellow arrow (injection site) indicates the plane between the epimysial layers within the temporalis. With a 27 G 38 mm needle, 1–2 mL of saline is injected via the out-of-plane approach. The inset (bottom right) shows the patient position and probe placement.

• Anatomy: From the temporal fossa to the coronoid process of the mandible.
• Referred pain: Maxillary molars, temporal headache, frontal region, periorbital region.
• Ultrasound: A linear probe is placed over the temporal region above the zygomatic arch to image the temporalis in the short axis.
• US-FHR: 27–30 G needle, 1–2 mL into the muscle belly and the fascia at the coronoid process insertion (Figure 3).

POINT 3: Lateral pterygoid/fat pad/perivascular maxillary artery/pterygopalatine region (integrated POINT)

The lateral pterygoid has a superior and an inferior head and is the prime mover of mouth opening and lateral mandibular movement. Between the deep layer of the temporalis and the superficial aspect of the lateral pterygoid, there is a continuous fascia between the two muscles containing the fat pad and the maxillary artery (running toward the pterygopalatine ganglion). With a single linear-probe approach, multiple adjacent structures are simultaneously addressed; this is the conceptual core of the integrated POINT.

**Figure 4:**
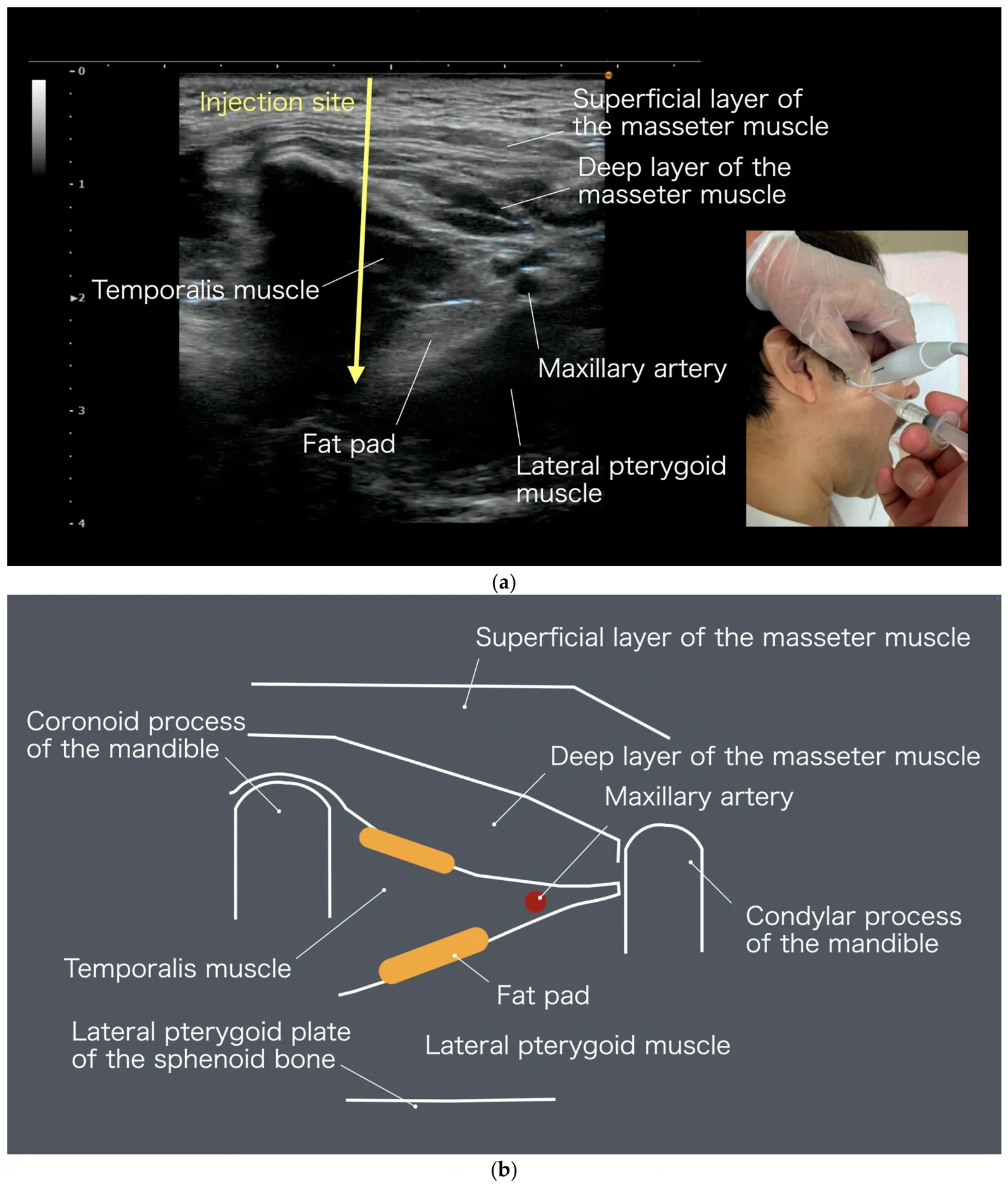
Integrated US-FHR of the lateral pterygoid/fat pad/maxillary artery/pterygopalatine region (POINT 3). (**a**) Ultrasound image: Superficial and deep layers of the masseter, temporalis, lateral pterygoid, fat pad, and maxillary artery are identified; the injection plane is directed toward the pterygopalatine fossa, which itself is not directly visualized. The yellow arrow (injection site) indicates the integrated target plane reached through the space between the superficial and deep epimysiums of the masseter. Color Doppler is mandatory to confirm the maxillary artery before injection. (**b**) Anatomical schema of the lateral pterygoid region (illustration by Ayato Kurosawa/Kimura Pain Clinic). In (**a**), the inset (bottom right) shows the patient position and probe placement.

• Anatomy: Fascia and fat pad between the deep layer of the temporalis and the superficial aspect of the lateral pterygoid and the maxillary artery (running toward the pterygopalatine ganglion); from the lateral pterygoid plate to the articular disk and the condylar process.
• Referred pain: Periarticular region of the TMJ, deep ear pain, occlusal pain, maxillary molars, maxillary sinus and nasal area, and autonomic-related symptoms.
• Ultrasound: A linear probe is placed over the cheek inferior to the zygomatic arch; after confirming the temporalis and the coronoid process, the deep lateral pterygoid, fat pad, and maxillary artery are imaged in the short axis. Color Doppler identification of the maxillary artery is mandatory for safety.
• US-FHR: 30 G needle, 1–2 mL into the fat pad between the lateral pterygoid and the deep masseter, and into the perivascular fascia of the maxillary artery. After confirming the maxillary artery with color Doppler, Doppler is turned off to maintain clear B-mode visualization during the release (Figure 4).

### 6.2. Medial Pterygoid and Joint Capsule Region (POINTs 4–6)

POINT 4: Medial pterygoid (extraoral approach)

**Figure 5:**
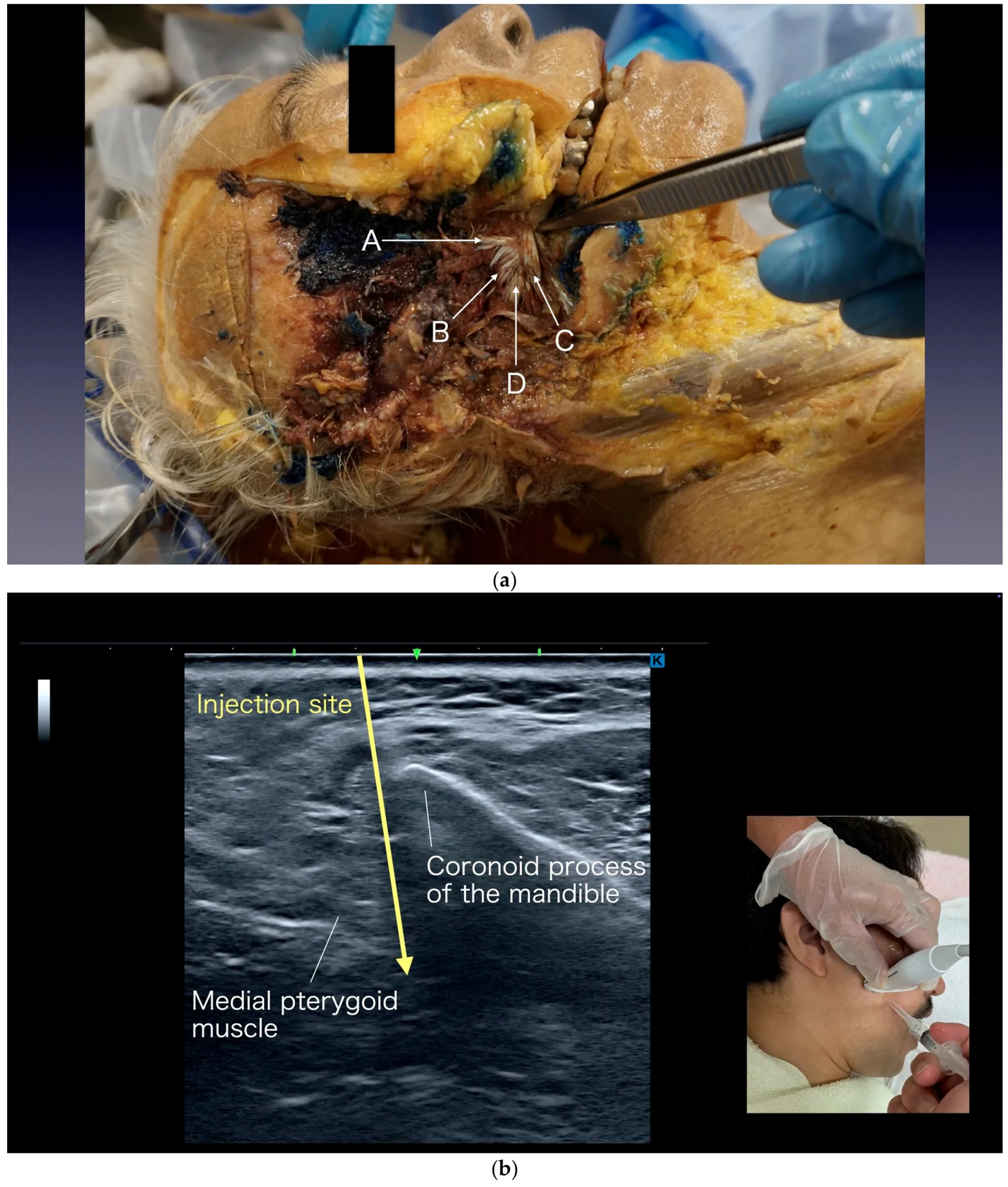
Anatomical rationale, US-FHR ultrasound image, and external view of the procedure for the medial pterygoid/fat pad (POINT 4). (**a**) Cadaveric dissection (photographed during the anatomy course at Nihon University School of Medicine on 30 November 2017; Nihon University Ethics Committee 28-8-0 approval number; corresponds to Figure 2a in our previous report [3]). Labels: A, temporalis muscle; B, inferior head of the lateral pterygoid muscle; C, deep head of the medial pterygoid muscle; D, superficial head of the medial pterygoid muscle (privacy masking applied). (**b**) Ultrasound image at the medial pterygoid region; inset shows the external view of the extraoral approach from anterior to the coronoid process under wide mouth opening.

• Anatomy: The medial pterygoid arises from the maxilla and from both surfaces of the lateral pterygoid plate, inserting on the medial surface of the mandibular ramus. Referred pain involves the periarticular region of the TMJ, mandibular molars, medial cheek, tongue, and palate. The fascia around the masseter and medial pterygoid—and the fat pad between them—are regarded as the putative pain generator. Insertion points of the temporalis, lateral pterygoid, and medial pterygoid are shown in Figure 5 (Nihon University Ethics Committee approval number 28-8-0).
  Precise correspondence of insertion sites:
  - Temporalis: Both surfaces of the coronoid process and the lateral pterygoid plate.
  - Lateral pterygoid: Surface and posterior margin of the lateral pterygoid plate.
  - Medial pterygoid: Maxilla and both surfaces of the lateral pterygoid plate.

This corresponds to POINT 2 (medial pterygoid/fat pad) in our previous article [3]. To contrast it with POINT 5 (intraoral approach), in the present article, this POINT is explicitly designated as the extraoral approach.

• Referred pain: Temporomandibular joint region, mandibular molars, medial cheek, tongue, and palate. In the authors’ clinical experience, this POINT is also selected for periarticular TMJ pain.
• Ultrasound: With the mouth half-opened and a convex probe, a panoramic ultrasound view facilitates the identification of the relevant structures. The patient positioning and imaging procedure is as follows:
  ① Lateral decubitus position with the affected side upward. The operator stands on the patient’s dorsal side.
  ② Image the temporalis in the short axis using the same imaging approach as for the temporalis/lateral pterygoid/masseter (Figure 3 and Figure 4 in the present article).
  ③ Slide the probe slightly caudally to display the bony outline of the coronoid process of the mandible.
  ④ From the oral side of the coronoid process, the structures appear from superficial to deep as follows: masseter, fat pad, and medial pterygoid.
  ⑤ While imaging with ultrasound, palpate intraorally posterior to the back molars toward the maxilla; medial pterygoid movement can be confirmed.
• US-FHR: Release the space between the medial pterygoid and the fat pad, or the fat pad itself (step ⑥).
  - Needle: 27 G 38 mm (using the needle length for the extraoral approach; out-of-plane).
  - Target: The space between the medial pterygoid and the fat pad, or the fat pad itself.
  - Injection volume: 1–2 mL (mainly physiological saline).
  - Injection site: Indicated by the yellow arrow.
  - Release area: Indicated by the yellow dashed line (the fat pad and the surrounding fascia).

The following are clinical key points (in addition to the standard procedure of [3], Steps ①–⑥):

- Have the patient open the mouth widely. This shifts the mandible downward and enlarges the space between the coronoid process and the maxilla, securing a safe access route to the medial pterygoid.
- Insert the needle from anterior to the coronoid process. This enables a safe and efficient needle approach to the superficial aspect of the medial pterygoid and the fat pad.
- These additional clinical tips extend the standard procedure and contribute to safety and efficiency.

POINT 5: Medial pterygoid (intraoral approach)—newly added

The medial pterygoid can also be approached intraorally, particularly in cases of limited mouth opening or pronounced deep fascial densification, where the intraoral approach is advantageous. This POINT is not described in our previous article [3] and was conceived by co-author Dr. Tadashi Kobayashi (Development of Community Healthcare, Hirosaki University Graduate School of Medicine). The anatomical landmarks for ultrasound-guided intervention to the lateral pterygoid muscle have also been detailed by Bae et al. (2025) [20] and Lee et al. (2024) [21] as safety zones for botulinum toxin injection.

**Figure 6:**
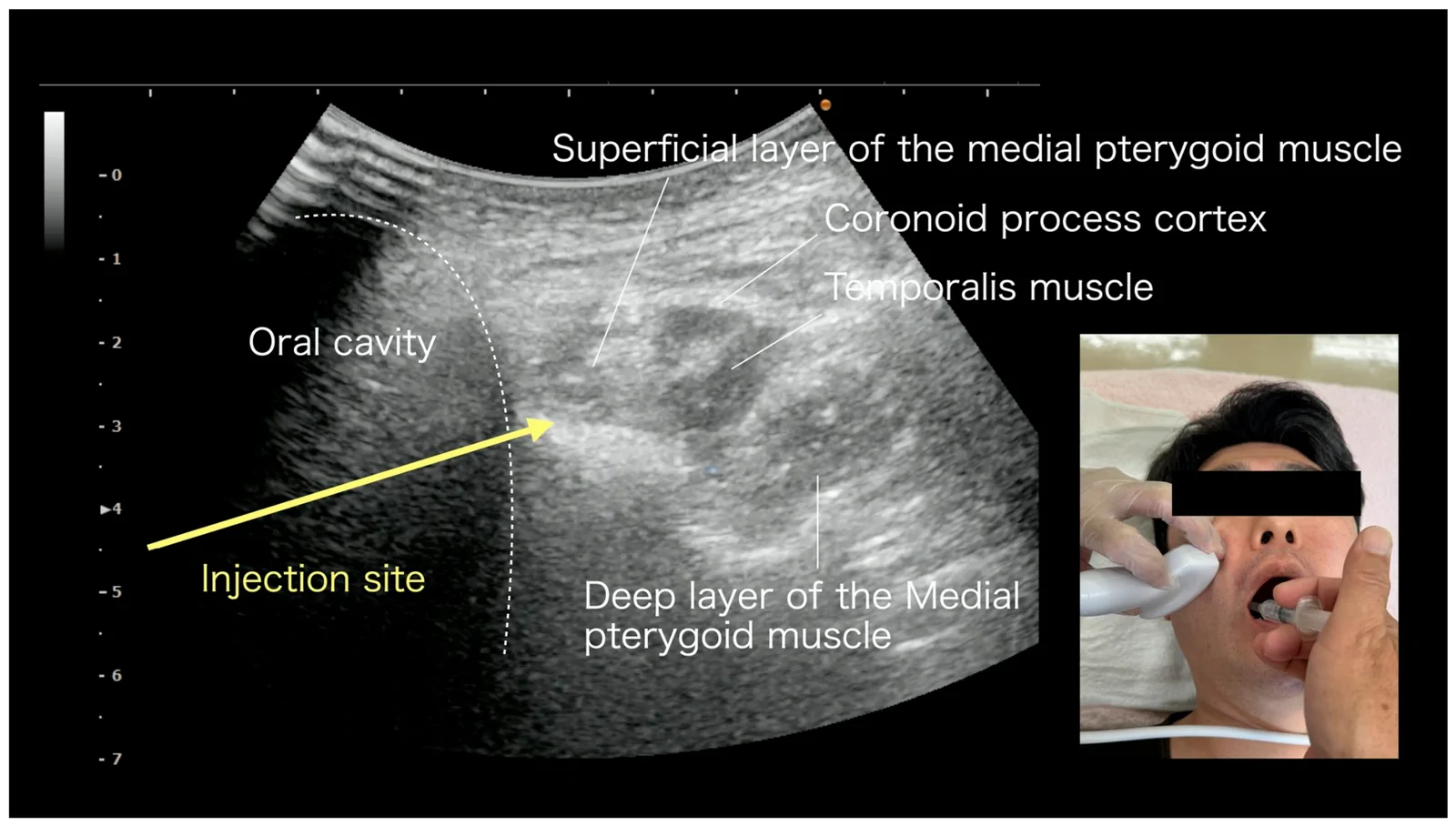
Medial pterygoid intraoral approach US-FHR (POINT 5). Ultrasound image: the oral cavity, the superficial and deep layers of the medial pterygoid, the coronoid process cortex, and the temporalis are identified. The yellow arrow (injection site) indicates the intraoral approach to the perimysial fat pad surrounding the medial pterygoid. The inset (bottom right) shows the patient position and probe placement.

• Anatomy: From the intraoral approach, posterior to the maxillary tuberosity, directly into the medial pterygoid muscle belly.
• Referred pain: The same as the extraoral approach.
• Indications: Limited mouth opening, pronounced deep fascial densification, cases complicated by contracture of the lateral pterygoid.
• US-FHR: 30 G needle, 0.5–1 mL via the intraoral route. Collaboration with a dentist is essential for safe execution (Figure 6).

POINT 6: Temporomandibular joint capsule complex

The temporomandibular joint capsule complex consists of the fibrous joint capsule (including ligamentous components) and the muscular insertions such as the lateral pterygoid, which are anatomically continuous without distinct tissue boundaries. As with the shoulder joint capsule, this POINT is treated as a unified structural complex, with the operator releasing this entire complex as a single target. Referred pain and dysfunction related to the temporomandibular joint are assessed with reference to the DC/TMD diagnostic criteria [14].

**Figure 7:**
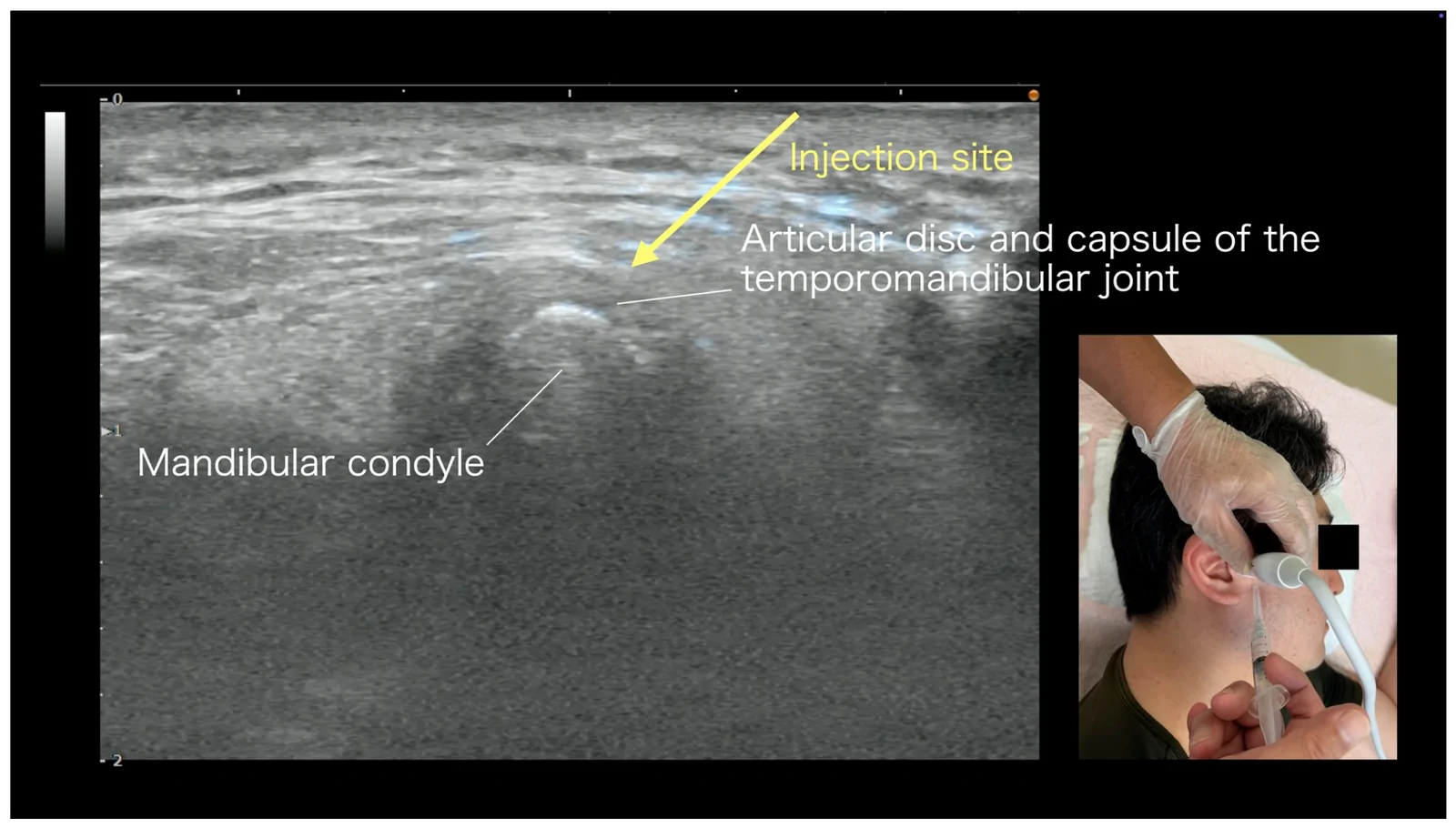
Temporomandibular joint capsule complex US-FHR (POINT 6). Ultrasound image: The mandibular condyle, articular disk, and joint capsule of the temporomandibular joint are identified. The yellow arrow (injection site) indicates the periarticular fascial space. The inset (bottom right) shows the patient position and probe placement.

• Anatomy: Inferior to the zygomatic arch; condylar process of the mandible, articular disk, joint capsule, and lateral pterygoid insertion.
• Referred pain: Periarticular TMJ region, pain on opening/closing, and occlusal discomfort.
• Ultrasound: Probe perpendicular to the zygomatic arch; the condylar process is tracked by sliding the probe, and joint dynamics are confirmed during opening and closing.
• US-FHR: 27–30 G needle, 1–2 mL into the densified periarticular fascia (Figure 7).

### 6.3. Parotid Region (POINT 7)

POINT 7: Superficial parotid fascia

US-FHR of the superficial parotid fascia is an intervention site first proposed as a treatment target by the authors in the present protocol; it was not included in our previous article [3]. The parotid gland forms a contiguous SMAS (superficial musculoaponeurotic system) layer from the superficial aspect of the masseter to the mastoid process, and the superficial parotid fascia is traversed by branches of the facial nerve, the parotid duct, and the transverse facial artery/vein.

Through clinical experience, densification of the superficial parotid fascia has been observed in patients with (1) non-odontogenic toothache, (2) temporomandibular disorders, (3) referred pain to the submandibular and cervical regions, (4) long-standing facial nerve palsy, post-facial-palsy synkinesis, (5) atypical facial pain, and (6) tinnitus. Of these, categories (4) to (6) represent exploratory clinical observations; they are not indications of the present protocol.

**Figure 8:**
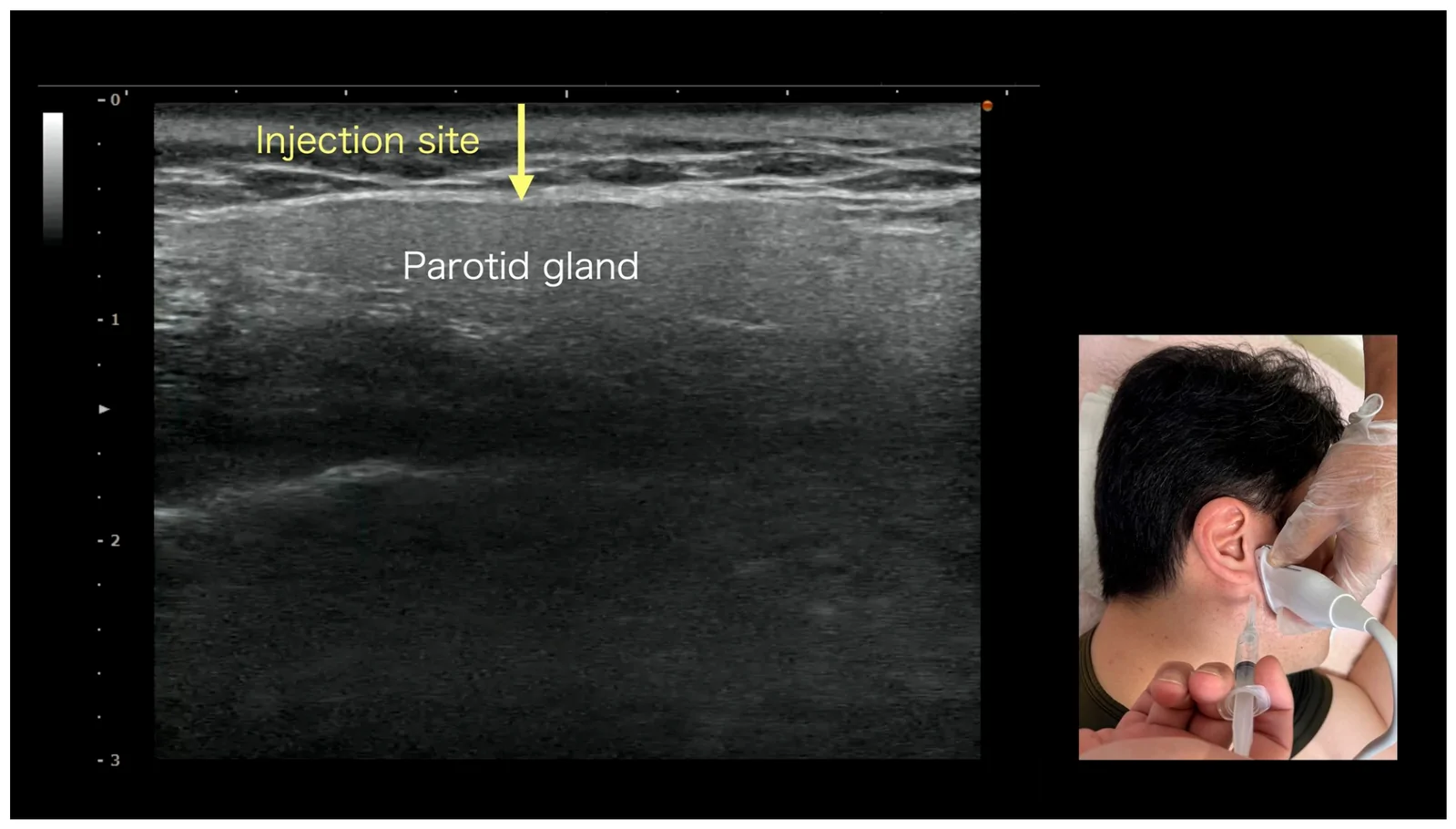
Superficial parotid fascia US-FHR (POINT 7). Ultrasound image: The parotid gland is identified. The yellow arrow (injection site) points to the densified area of the superficial parotid fascia. With a 30 G needle, 0.5–1 mL of saline is injected via the out-of-plane approach. The needle tip is kept superficial to the parotid gland capsule. The inset (bottom right) shows the patient position and probe placement.

• Anatomy: From the zygomatic arch to the anteroinferior region of the mastoid process to the posterior border of the mandibular ramus; continuous SMAS layer.
• Referred pain: Dental pain in general (low site specificity), periarticular TMJ region, cheek, and preauricular region.
• Ultrasound: The probe is moved caudally from the preauricular region; the parotid gland and the superficial layer of the masseter are identified, and the capsule layer is visualized.
• US-FHR: 30 G needle, 0.5–1 mL into the superficial parotid capsule layer; the facial artery and posterior auricular artery must be avoided (Figure 8).
• Related reports: Detailed extensions will be addressed in a separate case report (applications to long-standing facial nerve palsy, synkinesis, atypical facial pain, tinnitus, etc.).

### 6.4. Cervical Region (POINTs 8–9)

POINT 8: Digastric muscle

The digastric muscle is a two-bellied muscle in which the anterior and posterior bellies are connected to the hyoid bone through an intermediate tendon. It elevates the hyoid bone during swallowing and contributes to mouth opening. The anterior belly is an important landmark of the submandibular triangle, and fascial abnormalities here can produce referred pain to the mandibular anterior region, the tongue, and the pharynx.

**Figure 9:**
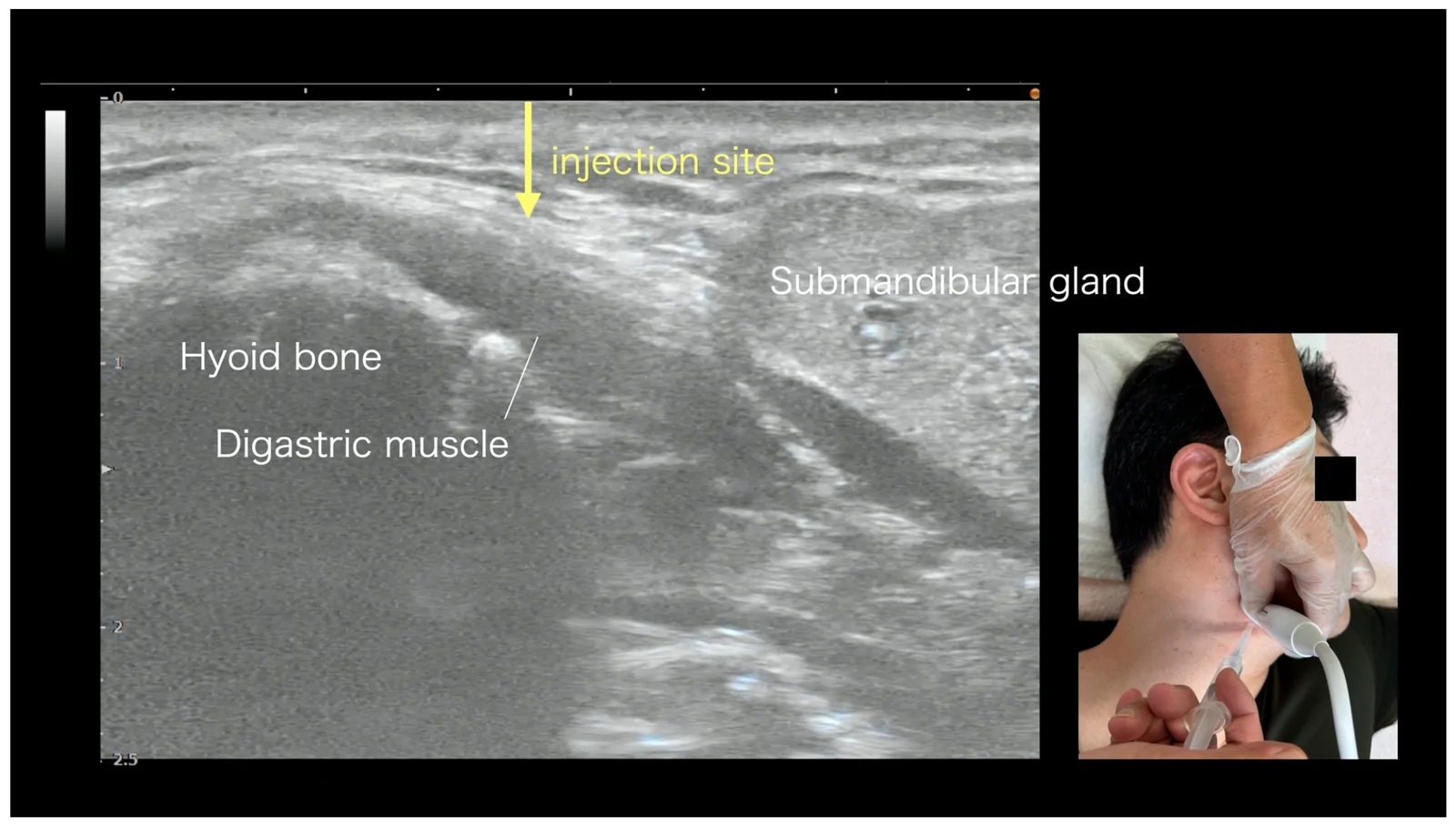
Digastric muscle US-FHR (POINT 8). Ultrasound image: Hyoid bone, digastric muscle, and submandibular gland are identified. The yellow arrow (injection site) indicates the fascia adjacent to the digastric muscle. With a 27 G 38 mm needle, 1–2 mL of saline is injected via the out-of-plane approach. The inset (bottom right) shows the patient position and probe placement.

• Anatomy: From the mental region to the hyoid bone to the mastoid process.
• Referred pain: Mandibular anterior region, tongue, pharynx, and swallowing pain.
• Ultrasound: Probe placed in the submental region; the hyoid bone and the submandibular gland serve as landmarks.
• US-FHR: 27–30 G needle, 1–2 mL into the fascia around the anterior belly and the intermediate tendon (Figure 9).

POINT 9: Cervical sympathetic ganglion region

Release around the cervical sympathetic ganglion region is a further development of POINT 4 (sternocleidomastoid/levator scapulae) from our previous article [3]. Through accumulated clinical experience, the present POINT was expanded to an integrated approach addressing—in addition to the sternocleidomastoid and levator scapulae—the scalene and longus colli muscles together with the perivascular tissue surrounding the cervical sympathetic trunk, centered on the middle cervical ganglion and extending cephalad and caudad to include the superior cervical and stellate ganglia. The safety principles established for ultrasound-guided interventions in this region—including identification of vascular and soft-tissue structures before needle advancement and precise fascial-plane targeting anterolateral to the longus colli—have been described for stellate ganglion block [22], and the Doppler-based procedure of the present protocol follows the same principles.

**Figure 10:**
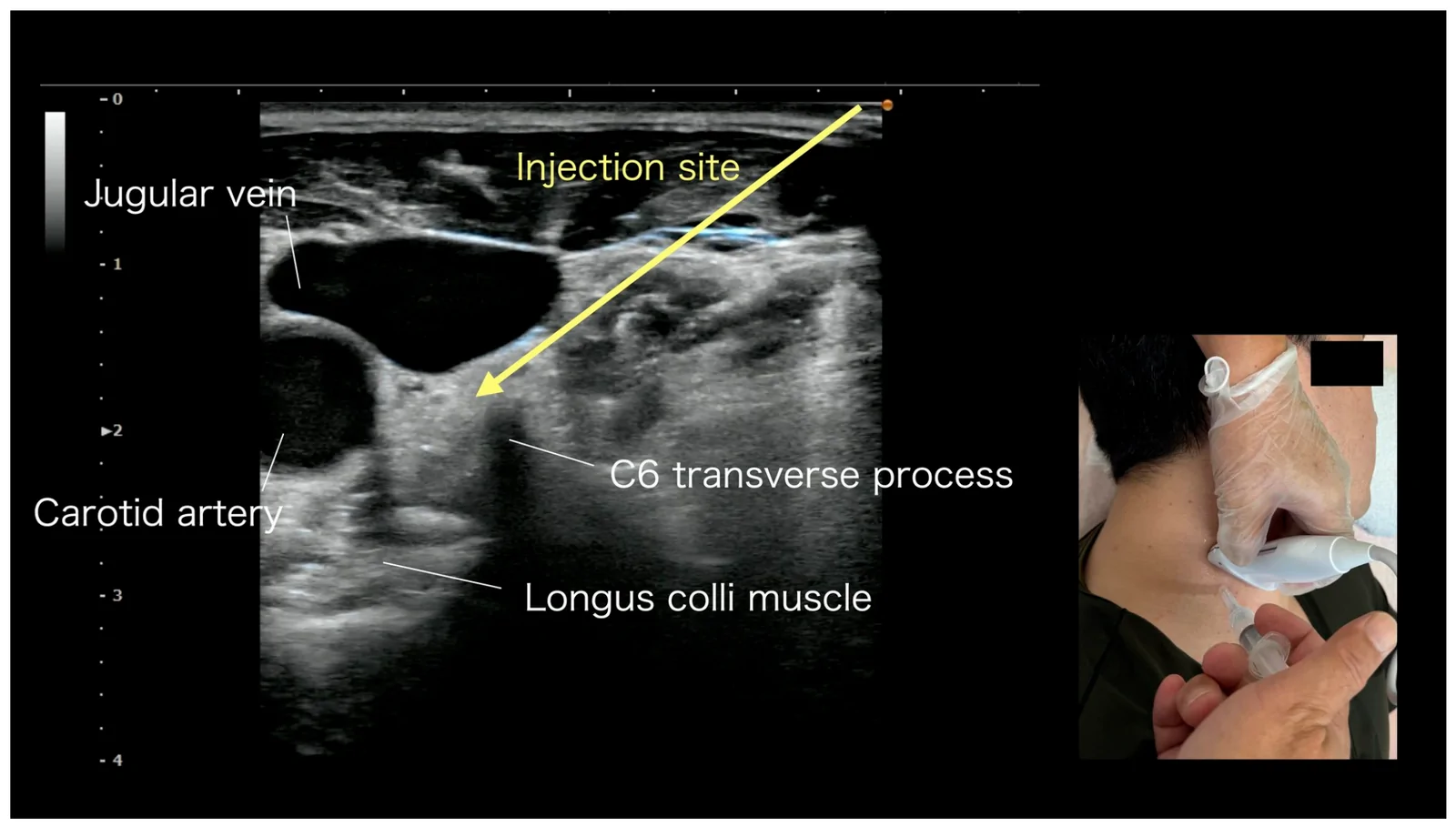
US-FHR around the cervical sympathetic ganglion region (centered on the middle cervical ganglion, extending cephalad and caudad to include the superior cervical and stellate ganglia) (POINT 9). Ultrasound image: Internal jugular vein, common carotid artery, and longus colli muscle are identified. The yellow arrow (injection site) indicates the perivascular fascial space surrounding the cervical sympathetic trunk. Color Doppler identification of vascular structures is mandatory for safety; Doppler is turned off during the release. The inset (bottom right) shows the patient position and probe placement.

• Anatomy: Fascial planes between the epimysiums of the sternocleidomastoid, levator scapulae, scalene, and longus colli muscles, and the perivascular sheath region.
• Referred pain: Temporal/frontal headache, periorbital pain, dizziness, tinnitus, and swallowing discomfort.
• Ultrasound: Palpate the sternocleidomastoid, place the probe at the tender point, and confirm the internal jugular vein and common carotid artery.
• US-FHR: 27–30 G needle, 1–2 mL into the densified fascia lateral to the carotid sheath (a slightly larger volume than other POINTs is used to ensure spread across multiple cervical fascial planes—between the epimysiums of the sternocleidomastoid, levator scapulae, scalene, and longus colli—and the perivascular fascia). After confirming vascular structures with color Doppler, Doppler is turned off to maintain clear B-mode visualization during the release (Figure 10).

### 6.5. Facial Artery Region (POINT 10)

POINT 10: Perivascular fascia of the facial artery

The facial artery crosses the mandibular border and runs obliquely over the buccinator and deep to the platysma. Fascial abnormalities around the artery are hypothesized to cause referred pain to the cheek, the corner of the mouth, the lower lip, and the mandibular anterior teeth. The concept of perivascular fascial release is presented here as a newly proposed concept of the present protocol.

**Figure 11:**
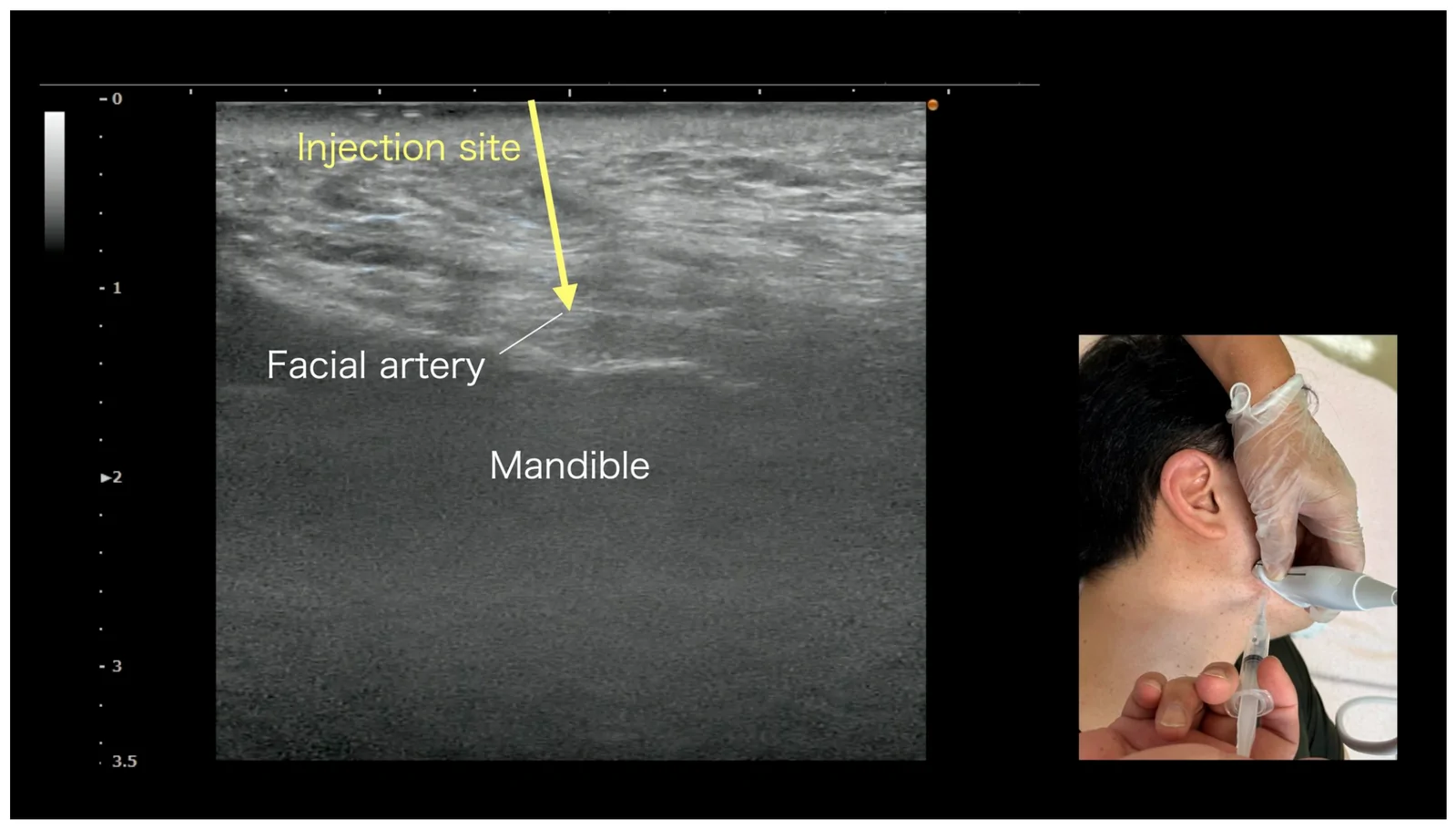
Perivascular fascia of the facial artery US-FHR (POINT 10). Ultrasound image: facial artery and mandible are identified. After confirmation of the artery with color Doppler, the yellow arrow (injection site) indicates the fascia lateral to the arterial sheath. A small volume is injected. Color Doppler identification of the artery is mandatory for safety; Doppler is turned off during the release. The inset (bottom right) shows the patient position and probe placement.

• Anatomy: From the inferior mandibular border to the superficial buccinator to the deep platysma.
• Referred pain: Cheek, corner of the mouth, lower lip, mandibular anterior teeth, and mental region.
• Ultrasound: Probe placed at the mandibular border; color Doppler identification of the facial artery.
• US-FHR: 30 G needle, 0.5–1 mL into the fascia lateral to the arterial sheath. After confirming the facial artery with color Doppler, Doppler is turned off to maintain clear B-mode visualization during the release (Figure 11).

### 6.6. Upper Posterior Cervical Release (POINT 11)—Advanced Technique (C0–1 Vertebral Artery Curvature or C1–C2 Ligamentum Flavum/Dural Region; Selected by Tender-Point Dominance)

This section describes an advanced technique that the authors have applied to refractory non-odontogenic toothache projecting from the upper teeth to the retro-orbital and temporal regions.

POINT 11: Upper posterior cervical release (integrated POINT, Advanced)
Three structures simultaneously addressed by POINT 11

POINT 11 is not merely a densification release of the PAOM/dura/ligamentum flavum. In a single procedure, the following three structures are simultaneously addressed:

(1) PAOM/dura densification release: The C0–1 posterior atlanto-occipital membrane and the C1–C2 ligamentum flavum/epidural space are histologically continuous [23], and the densification along this continuum is released through a posterior, out-of-plane needle approach. The target is the ligamentum flavum itself (C1–C2) or the posterior atlanto-occipital membrane (C0–1); spread into the epidural space may occur but is not the intended target. Bone contact with the C1 posterior arch is avoided, because a needle tip burred by bone contact can cause tissue injury.
(2) Densification release at the vertebral artery curvature: After passing through the C1 transverse foramen, the vertebral artery makes a sharp curve on the superior surface of the C1 posterior arch. This curvature corresponds to category #1 (curved regions of tissues) among the eight stacking-fascia predilection categories [12] and is a site where densification has been observed in the authors’ clinical experience.
(3) Perivascular fascia release around the vertebral artery: This step requires particular attention. It addresses densification of the perivascular fascia around the vertebral artery (stacking-fascia category #6, superficial course of neurovascular structures [12]). Whether this release produces changes in vertebral arterial hemodynamics that, in turn, affect the perfusion of the brainstem, cerebellum, and upper cervical spinal cord is a hypothesis that remains to be tested by direct flow measurement; no direct flow measurement was performed in the present work. This aspect supports a hypothesized rationale for applying POINT 11 to non-odontogenic toothache, particularly in the maxillary molar region; further prospective evaluation is required.

In our clinical observations, in patients presenting with occipital pain, dizziness, tinnitus, or maxillary molar referred pain, POINT 11—used either alone or in combination with other POINTs—has been associated with symptomatic improvement. These observations are not fully explained by the densification release of the PAOM/dura/ligamentum flavum alone; perivascular release around the vertebral artery and its potential effect on arterial hemodynamics may also be considered as a contributing factor, although this requires further investigation.

**Figure 12:**
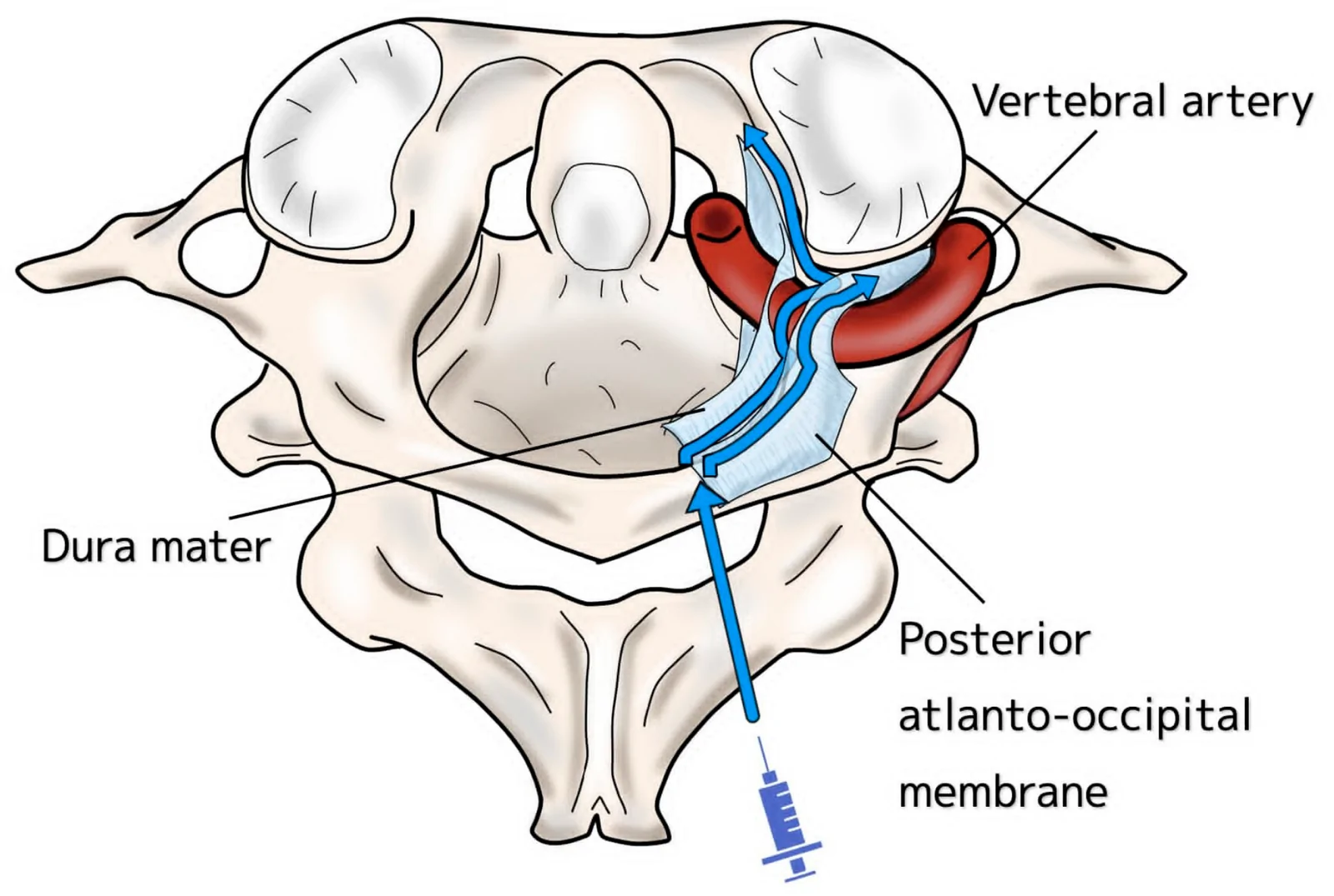
Anatomical basis for upper posterior cervical release: histological continuity of the PAOM, dura, and ligamentum flavum, and integration with the perivascular fascia of the vertebral artery (common to POINT 11; illustration by Hidenori Horigome/Kimura Pain Clinic). Axial view of the upper cervical spine seen from the superior aspect of the C1 arch. The vertebral artery (red) passes through the C1 transverse foramen, makes a sharp curve on the superior surface of the C1 posterior arch, and runs superficially relative to the posterior atlanto-occipital membrane (PAOM). The PAOM and the dura mater are histologically continuous [23]; the perivertebral fascia at C0–1 and the ligamentum flavum/epidural space at C1–C2 can be accessed via different levels of this posterior membranous structure. The blue arrow indicates the posterior out-of-plane needle approach. Depending on tender-point dominance, the C0–1 (POINT 11A; Figure 13) or C1–C2 (POINT 11B; Figure 14) level is selected, and three structures are simultaneously addressed in a single procedure: (1) histological continuity of the PAOM and dura, (2) densification of the perivascular fascia at the vertebral artery curvature on the superior surface of the C1 posterior arch, and (3) the ligamentum flavum and epidural space at C1–C2. The essence of the procedure is densification release of the PAOM, dura, and ligamentum flavum, together with perivascular release around the vertebral artery; whether this perivascular release influences vertebral arterial hemodynamics and, in turn, perfusion of the brainstem, cerebellum, and upper cervical spinal cord is a hypothesis that remains to be tested by direct flow measurement. As indicated by the blue arrows in this schematic, the injectate is distributed across the following structures: the PAOM or the ligamentum flavum itself, the epidural space, the perivascular region around the vertebral artery, and the tissues anterior to it.

As shown in Figure 12, the injectate is distributed across the following structures: the PAOM or the ligamentum flavum itself, the epidural space, the perivascular region around the vertebral artery, and the tissues anterior to it. On palpation, the C0–1 (vertebral artery curvature and posterior atlanto-occipital membrane) and C1–C2 regions (ligamentum flavum) are evaluated, and the side with the more prominent tenderness is selected as the target. A single posterior needle approach is used to distribute the injectate across the structures described above. POINT 11 is considered by the authors only in refractory pain extending from the upper teeth through the retro-orbital region to the temporal region, accompanied by tenderness in the upper posterior cervical area.

**Figure 13:**
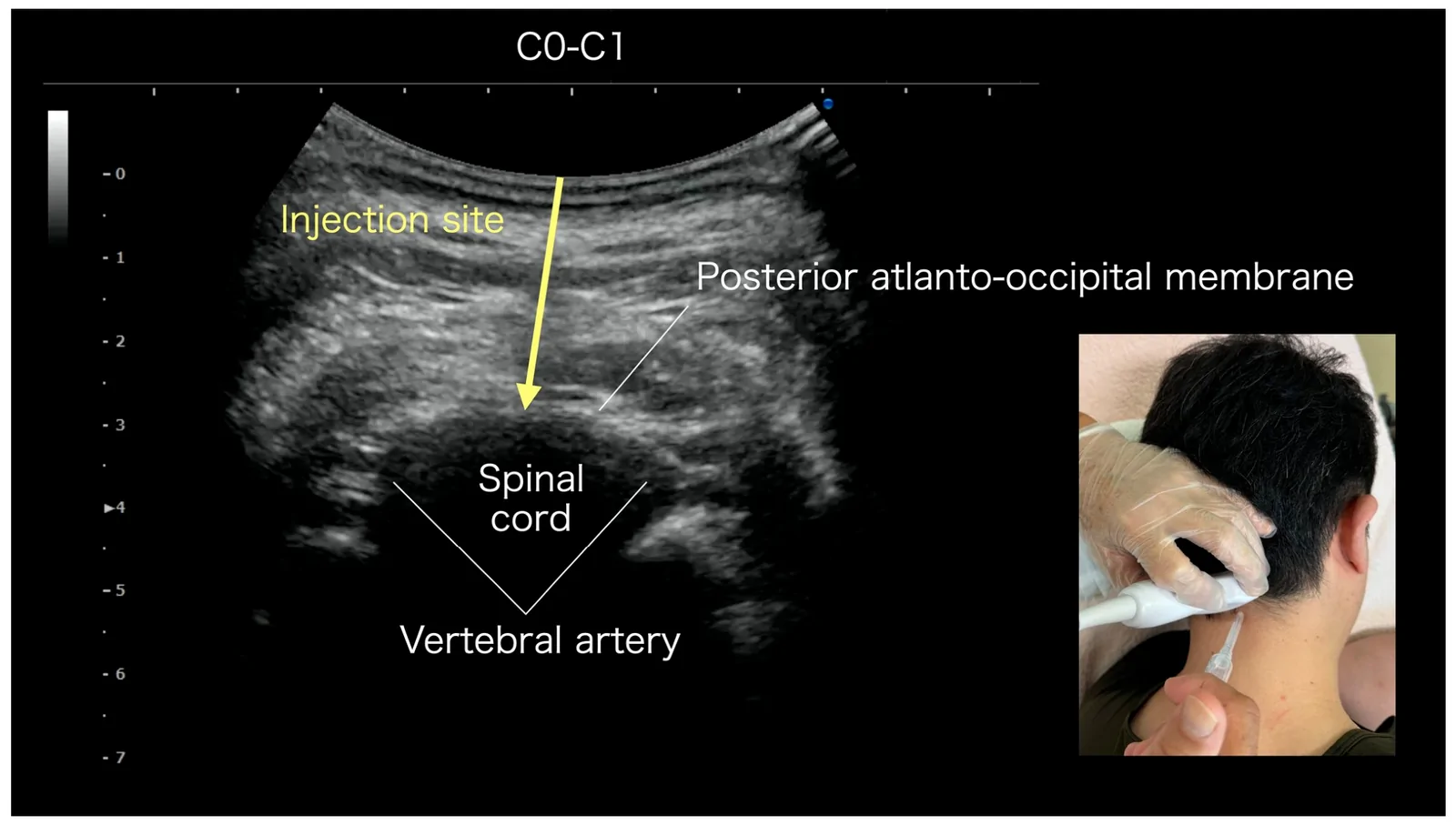
Upper posterior cervical release at C0–1 (POINT 11A, vertebral artery curvature; Advanced). Ultrasound image: Posterior atlanto-occipital membrane (PAOM), vertebral artery, and spinal cord are identified. The yellow arrow (injection site) indicates posterior advancement to the PAOM and release of the perivascular fascia around the vertebral artery curvature, together with densification along the PAOM. Selected in patients with dominant tenderness in the C0–1 region. The inset (bottom right) shows the patient position and probe placement.

**Figure 14:**
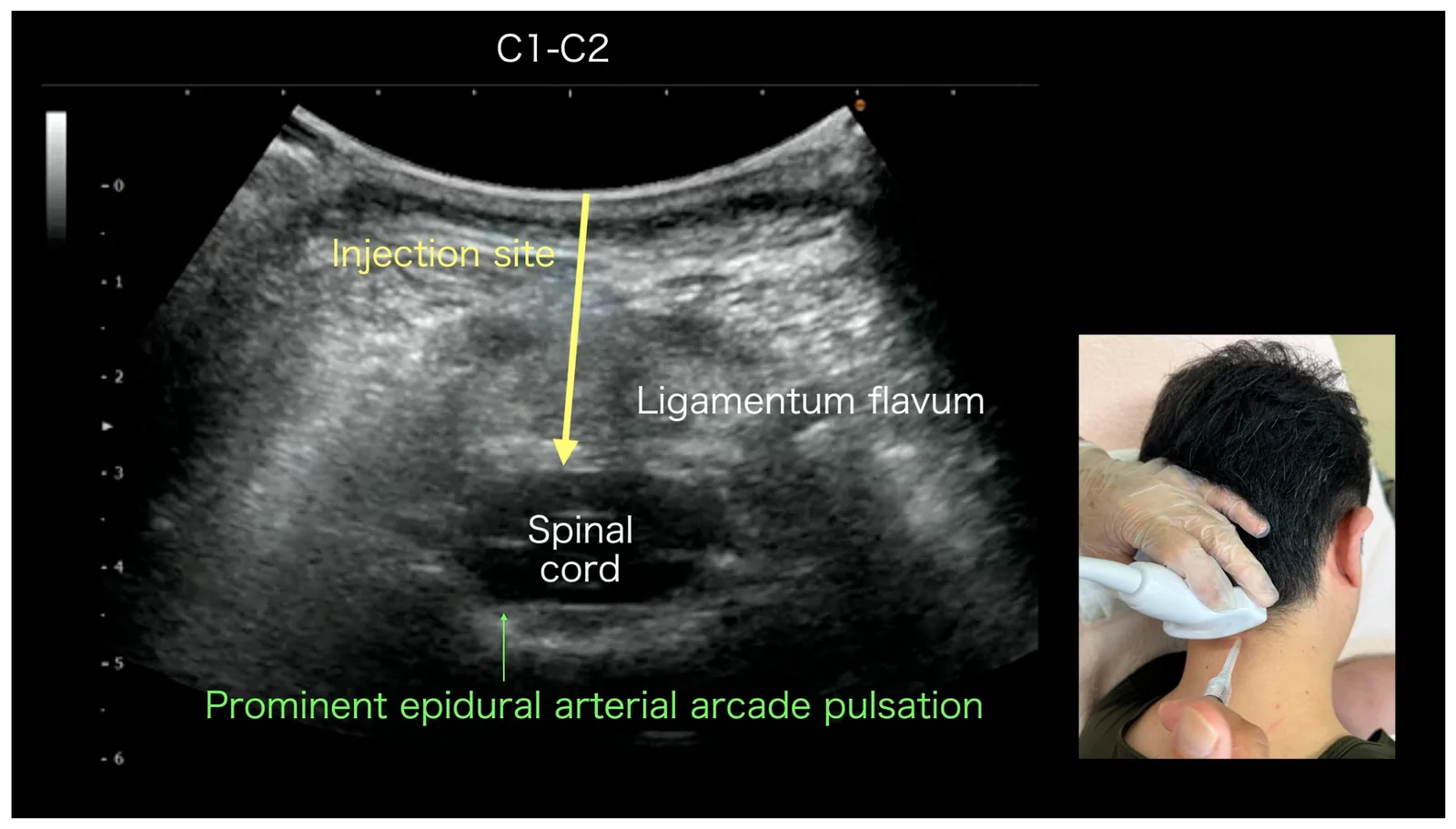
Upper posterior cervical release at C1–C2 (POINT 11B, ligamentum flavum and dural region; Advanced). Ultrasound image: Ligamentum flavum, spinal cord, and prominent epidural arterial arcade pulsation are identified. The yellow arrow (injection site) indicates posterior advancement to the ligamentum flavum/dural region and densification release of the PAOM, ligamentum flavum, and epidural space. Selected in patients with dominant tenderness in the C1–C2 region. POINT 11A and POINT 11B together constitute a single procedure selected according to tender-point dominance, and the two are treated as a continuous posterior cervical structure addressed in a unified manner. The inset (bottom right) shows the patient position and probe placement.

• Anatomy: At C0–1, the perivascular fascia around the vertebral artery at the C0–1 curvature segment (beneath the posterior atlanto-occipital membrane), or at C1–C2, the ligamentum flavum and dura mater. The two structures are histologically continuous [23].
• Referred pain: Refractory pain from the upper teeth (especially the maxillary molars) through the retro-orbital region to the temporal region; refractory headache; and cases with prominent upper posterior cervical tenderness.
• Ultrasound: Based on palpation, the side with dominant tenderness (C0–1 or C1–C2) is selected, and a convex probe (C5-2) is used to image the corresponding level. Color Doppler identification of the vertebral artery is mandatory for safety.
• US-FHR: 30 G needle, advanced from the posterior side, with the tip placed beneath the PAOM; 1–2 mL of physiological saline is injected (direct vascular puncture is strictly forbidden; color Doppler identification of the vertebral artery is mandatory; Doppler is turned off during the release).
• Treatment Selection Algorithm: The side with dominant tenderness (C0–1, Figure 13; or C1–C2, Figure 14) is selected first; if both are equivalent, C0–1 (more superficial and safer) is preferred.

Advanced Warning: POINT 11 is classified as Advanced; the operator, supervision, and facility requirements specified in Table 1 apply. Because of the risk of vertebral artery injury, it should only be performed by operators fully trained in neurovascular ultrasound anatomy. Direct intravascular puncture must be strictly avoided, and injection should be limited to the surrounding fascial layers.

## 7. Pathology-Specific Application of the Eleven-Point Protocol

This chapter describes the application strategies of the eleven-point protocol for representative pathological and anatomical patterns of non-odontogenic toothache and orofacial pain. The main POINTs for each category are applied first, and adjunctive POINTs are added when the response is insufficient.

### 7.1. Masticatory Myofascial Toothache

Non-odontogenic toothache arising from trigger points and fascial densification of the masseter, temporalis, lateral pterygoid, and medial pterygoid. Main POINTs: 1–5. Adjunctive POINTs: 6 (TMJ capsule complex) and 7 (superficial parotid). This category is common in the authors’ clinical practice and is consistent with the referred-pain patterns of trigger points in the masticatory and neck–shoulder muscles in patients with temporomandibular disorders, as classically described by Travell and Simons [7] and Fernández-de-las-Peñas (2010) [10].

### 7.2. Toothache Related to Perivascular Fascia (Newly Proposed)

Non-odontogenic toothache may be related, as a hypothesis, to perivascular fascial abnormalities (perivascular facial artery, deep maxillary artery, perivertebral vertebral artery). This category is distinct from neurovascular toothache as defined in the JSOP classification [1] and the ICOP [13] (toothache attributed to migraine or other primary headaches), which is excluded beforehand (Figure 1). Main POINTs: 3 (lateral pterygoid/fat pad/maxillary artery/pterygopalatine integrated POINT), 10 (perivascular fascia of the facial artery), and 11 (upper posterior cervical, Advanced). The protocol presents a clinical application of the perivascular fascial release concept proposed here. In support of this concept, a recently published prospective single-arm interventional study reported pain reduction with ultrasound-guided fascia hydrorelease around the artery for myofascial neck pain [24].

### 7.3. TMD-Related Toothache

Occlusal discomfort and dental pain are associated with dysfunction of the temporomandibular joint capsule complex and the lateral pterygoid. Main POINTs: 1 (masseter), 3 (lateral pterygoid/fat pad/maxillary artery), 4–5 (medial pterygoid), and 6 (TMJ capsule complex). The protocol is intended to address the pain sources commonly involved in TMD.

### 7.4. Autonomic-Component Toothache

Dental and orofacial pain with sympathetic hyperactivity originating from the cervical sympathetic ganglion region (centered on the middle cervical ganglion, extending cephalad and caudad to include the superior cervical and stellate ganglia). Main POINT: 9 (cervical sympathetic ganglion region). Adjunctive: POINT 3 integrated POINT (pterygopalatine ganglion region). Detailed extension will be reported in a separate paper on Crowned Dens Syndrome (CDS) focusing on the perivascular release approach.

### 7.5. Swallowing-Related Toothache

Dental pain accompanied by swallowing pain and lingual discomfort may be related to the digastric muscle. Main POINT: 8 (digastric).

### 7.6. Parotid-Derived Toothache (Newly Proposed)

Dental and facial pain hypothesized to arise from densification of the superficial parotid fascia is newly described in the present protocol. Main POINT: 7 (superficial parotid).

### 7.7. Refractory Referred Pain (Upper Teeth ↔ Deep Eye ↔ Temple)

Refractory referred pain may be related, as a hypothesis, to structural abnormalities of the upper posterior cervical region (C0–1 PAOM/C1–C2 ligamentum flavum). Main POINT: 11 (upper posterior cervical, Advanced). Through the Treatment Selection Algorithm, the dominant tender site (C0–1 vertebral artery curvature versus C1–C2 ligamentum flavum) is selected. POINT 11 is restricted to operators fully trained in neurovascular ultrasound anatomy.

## 8. Discussion

### 8.1. Overall Discussion

In this Discussion, the basis for each statement is distinguished according to four categories: established (direct) evidence, anatomical rationale (indirect evidence), expert clinical experience, and biological hypothesis. Statements are labeled accordingly throughout (e.g., “in the authors’ clinical experience,” “as a hypothesis”), and the same four-category framework is embodied in the evidence classification of Table 4 (Section 6).

The present article systematized the US-FHR (ultrasound-guided fascia hydrorelease) treatment regions for non-odontogenic toothache and related orofacial pain into an eleven-point protocol. The present protocol is the result of long-term refinement of the four sections at the corresponding author’s institution and is presented here as a shared anatomical language and educational framework.

In the present protocol, six new or significantly expanded POINTs were added: POINT 3 (integrated lateral pterygoid/fat pad/perivascular maxillary artery/pterygopalatine region), POINT 5 (medial pterygoid, intraoral approach), POINT 7 (superficial parotid fascia), POINT 9 (cervical sympathetic ganglion region, integrated approach), POINT 10 (perivascular fascia of the facial artery), and POINT 11 (upper posterior cervical release, Advanced). The arrangement into eleven POINTs aims to function as a shared vocabulary for pain physicians, dentists, physical therapists, and acupuncturists.

Notable newly added POINTs include the following:

• POINT 5 (intraoral medial pterygoid): Newly added; this approach was conceived by co-author Dr. Tadashi Kobayashi (Development of Community Healthcare, Hirosaki University Graduate School of Medicine).
• POINT 7 (superficial parotid fascia): In the authors’ clinical experience, symptomatic improvement has been observed in selected cases of non-odontogenic toothache; these observations are hypothesis-generating and require prospective evaluation.
• POINT 11 (upper posterior cervical release): An integrated technique that simultaneously addresses (1) the histological continuity of the PAOM and dura, (2) the densification at the vertebral artery curvature at C1, and (3) the perivascular fascia of the vertebral artery. Whether this is accompanied by measurable changes in vertebral arterial hemodynamics or downstream perfusion of the brainstem, cerebellum, and upper cervical spinal cord remains a hypothesis to be tested by direct flow measurement.

The protocol organizes the rationale for each POINT from the perspective that fascial pathological changes (densification, stacking, and impaired gliding) are clinically common contributors to such pain.

The authors acknowledge the diagnostic value of comparative diagnostic procedures such as local anesthetic blocks and controlled provocation testing in the assessment of orofacial pain. Because the present protocol deliberately uses physiological saline or bicarbonate Ringer’s solution without local anesthetic, such comparative diagnostic procedures are not part of the protocol itself; they are identified as an important element of future validation studies.

### 8.2. Conceptual Mapping to the Eight Stacking-Fascia Categories

The mapping of each POINT in the present protocol to the eight predilection categories of stacking fascia, as proposed in our prior Memory Reset Hypothesis paper [12], indicates that the intervention sites correspond to the major categories (#1 curved regions, #4 peritubular, #5 fat pads, #6 superficial neurovascular course, and #7 ligamentum flavum/epidural space). This correspondence is presented as a conceptual mapping demonstrating internal consistency with the authors’ published hypothesis framework [12], not as independent evidence of anatomical validity; independent validation remains pending.

The following POINT-specific composite mappings are particularly important:

• POINT 3 (lateral pterygoid/fat pad/maxillary artery/pterygopalatine) = #1 curved regions + #4 peritubular + #5 fat pad + #6 superficial neurovascular course (quadruple composite; integrated POINT).
• POINT 11 (upper posterior cervical) = #7 ligamentum flavum and epidural space (PAOM–dura histological continuity) + #1 curved regions (vertebral artery curvature) + #6 superficial neurovascular course (perivascular fascia of the vertebral artery) (triple composite; integrated POINT, addressing densification across the PAOM/dura/ligamentum flavum together with perivascular release of the vertebral artery; possible downstream effects on brainstem, cerebellar, and upper cervical spinal cord perfusion via altered vertebral arterial hemodynamics remain a hypothesis to be tested).
• POINT 7 (superficial parotid) = #6 superficial neurovascular course (the facial nerve and parotid duct travel beneath the superficial fascia).

The proposed mechanism underlying POINT 11 is consistent with recent evidence on the ligamentum flavum and PAOM as pain sources.

Traditionally, the ligamentum flavum was considered an elastic-fiber-rich structure with sparse innervation [25]. Recent immunohistochemical studies have shown that under pathological conditions, the ligamentum flavum can function as a putative pain generator.

In patients with lumbar spinal stenosis, Benditz et al. [19] demonstrated a significant increase in sensory nerve fiber density (sensory hyperinnervation) in the ligamentum flavum compared with controls, showing that nerve fiber density correlated positively with clinical pain indices and functional impairment [19].

Furthermore, through a histological study of the cervical ligamentum flavum (C3–C7), Wu et al. [26] reported the abundant distribution of sympathetic nerve fibers, providing an anatomical basis for cervical vertigo [26].

In addition, the PAOM is connected to the pain-sensitive dura mater via the myodural bridge [23], and tension here is known to elicit pain signaling originating from the dura. Furthermore, dural afferents at the upper cervical spinal cord converge onto the spinal trigeminal nucleus (trigeminocervical convergence) and may project as referred pain to the head and face. This mechanism supports the anatomical plausibility of POINT 11 in non-odontogenic toothache, particularly in the maxillary molar region, and suggests a possible neuroanatomical pathway for the clinical observations described above.

Taken together, POINT 11 may simultaneously address (1) the ligamentum flavum as a possible pain generator via sensory hyperinnervation, (2) potential dura-derived pain mediated by the PAOM–myodural bridge, (3) maxillary molar referred pain via trigeminocervical convergence, and (4) a possible effect on vertebral arterial perfusion via release of the perivascular fascia of the vertebral artery, suggesting a plausible—but still hypothetical—quadruple mechanism that requires prospective validation.

### 8.3. From TPI to FHR: A Complementary Anatomical Perspective

The present protocol proposes a complementary anatomical perspective, from intramuscular trigger-point injection (TPI) to fascia hydrorelease (FHR). Conventional TPI for non-odontogenic toothache and related orofacial pain syndromes targets the muscle belly itself, whereas the present US-FHR targets the space between epimysiums, the fat pad, and the perivascular fascia. This perspective is grounded in the loose connective tissue dysfunction theory advanced by Stecco and colleagues and in our previous cadaveric study [4], demonstrating wide spread between epimysiums of small injection volumes via the out-of-plane approach. The electrophysiological basis of fascial densification is consistent with the hyaluronan-LCT dysfunction theory of the Stecco group [27,28,29,30,31]. Furthermore, the efficacy of interfascial injection for myofascial pain syndrome has been demonstrated in prospective, randomized, double-blinded trials [32], providing controlled-trial-level support for the fascia-targeted paradigm adopted in the present protocol, although the eleven-point US-FHR protocol itself has not yet been evaluated in a controlled trial.

### 8.4. Position Relative to Existing Related Studies

The fundamental difference between the present protocol and that of Chen et al. [33] lies in the target tissue and treatment paradigm. In line with classical trigger-point injection (TPI), Chen et al. deliver the injectate directly into the muscle belly of the pterygoid muscles. In contrast, the present US-FHR protocol injects 1–2 mL of saline into the space between epimysiums, the fat pad, and the perivascular fascia. This corresponds to a transition from an intramuscular approach to a fascia-targeted approach.

The work of Chen et al. [33] demonstrated procedural feasibility in a water-phantom model; clinical efficacy in actual patients remains a future research question. By contrast, in a previous cadaveric study [4] the present authors showed that 1.0 mL of injectate spreads broadly along the plane between epimysiums (24.50 cm^2^ on the deep side and 18.82 cm^2^ on the superficial side) and that the out-of-plane (oblique) approach is the most accurate technique for such an injection.

Vas et al.’s [34] work on ultrasound-guided dry needling of the masticatory muscles for trigeminal neuralgia (TGN) partially overlaps with the present protocol, in that it targets the masticatory muscles (masseter, temporalis, medial pterygoid, lateral pterygoid, and digastric). However, the target disease in their study was TGN rather than non-odontogenic toothache, and their procedure was dry needling without fluid injection, in contrast to the fascia hydrorelease with saline presented here. The clinical effect (NRS 8.9 → 0.6) reported by Vas et al. suggests that ultrasound-guided intervention to the masticatory muscles may be effective for refractory pain in the trigeminal territory and may indirectly support the clinical relevance of the FHR approach for non-odontogenic toothache presented here.

The series of studies by the Stecco group [5,27,28,29,30,31] provided multi-dimensional verification for the anatomical basis of hyaluronan aggregation, densification, and fascia-targeted injection. These form the theoretical foundation of the present protocol, although none of them propose a direct clinical application protocol for non-odontogenic toothache or orofacial pain. The present article integrates the theoretical foundation of the Stecco group with the clinical insights of Vas et al., presenting an eleven-point US-FHR protocol for non-odontogenic toothache and orofacial pain as a proposal for future clinical evaluation.

Importantly, the present protocol introduces the concept of integrated POINTs. POINT 3 (lateral pterygoid/fat pad/perivascular maxillary artery/pterygopalatine region) addresses four adjacent structures in a single linear-probe procedure, while POINT 11 (upper posterior cervical release) individualizes PAOM, dura, and ligamentum flavum treatment by selecting the dominant tender site (C0–1 vertebral artery curvature versus C1–C2 ligamentum flavum). These integrations extend Chen’s individual-muscle approach by incorporating the functional connectivity among adjacent structures into the treatment concept.

### 8.5. Brief Notes on Related Areas

POINT 11 essentially functions as a vertebral perivascular fascia release and is anatomically adjacent to structures implicated in Crowned Dens Syndrome (the C1–C2 region and peri-odontoid tissues) and to the perfusion territory of the vertebral artery. The authors have encountered cases in which symptoms such as dizziness, tinnitus, occipital pain, and referred pain to the upper molar region were observed in this context; however, any application to Crowned Dens Syndrome remains a subject for future independent investigation and is not an indication of the present protocol. For reference, conventional C1–C2 steroid injection for Crowned Dens Syndrome has been reported [35]; the present technique differs mechanistically in that it targets fascial densification. In addition, perivascular release around the occipital and superficial temporal arteries may be of interest as a related area in the context of post-herpetic neuralgia, and perivascular fascia release of the facial artery (POINT 10) may be of interest as a related area in the context of synkinesis following long-standing facial nerve palsy.

The same principle—organizing region-specific fascial densification into a point-based US-FHR protocol—can be extended to other anatomical regions. The present eleven-point orofacial protocol is intended as one component of a broader effort to systematize US-FHR applications anchored in fascial densification across body regions.

### 8.6. Limitations

This article is a clinically grounded synthesis aimed at organizing the literature and clinical experience; it does not present quantitative data on case numbers or treatment outcomes. Quantitative clinical outcome evaluation is left to future prospective studies. The protocol’s development relies on the long-term clinical experience and patient observations at the corresponding author’s institution, and the bias due to the limited number of practitioners and case selection must be acknowledged. An exhaustive list of candidate points that were considered but excluded during protocol development was not compiled. Controlled clinical data and standardized outcome measures are likewise lacking for the eleven-point protocol itself. POINT 11 is an advanced, investigational technique, and this article alone must not serve as the basis for its adoption into clinical practice or education.

In addition, four further limitations should be made explicit. First, because this is a clinical protocol article rather than a systematic review, no formal evidence-quality or risk-of-bias assessment of the cited literature was undertaken; the descriptive four-category framework in Table 4 provides a transparent characterization of the supporting literature for each POINT but does not replace such an appraisal. Second, because no control group or patient-level outcome data are presented, treatment effects cannot be separated from placebo responses, natural course, or the effects of co-interventions. Third, no defined follow-up period is reported, so the durability of any clinical change remains unknown. Fourth, the generalizability of the protocol beyond the corresponding author’s institution is uncertain, and the scope of practice for the procedures described here differs across professions and jurisdictions; the protocol must be applied only within each practitioner’s legally defined scope of practice.

Within the scope of the corresponding author’s clinical experience, no serious adverse events have been observed in association with the procedures described here. As minor events, subcutaneous bleeding at the puncture site has occasionally been observed, and vasovagal reactions have occurred rarely; all resolved with observation alone. Two structural factors are considered to underlie this safety profile: (1) all procedures are performed under ultrasound guidance with continuous visualization of the needle tip, and (2) no local anesthetics are used (physiological saline or bicarbonate Ringer’s solution only), so that the risks of local anesthetic systemic toxicity and allergy are structurally absent. However, adverse events were not systematically recorded, and this experience cannot substitute for prospective safety data.

## 9. Conclusions

This study proposes an expert experience-based, eleven-point interventional framework for ultrasound-guided fascia hydrorelease (US-FHR) in non-odontogenic toothache and related orofacial pain. It is an experience-based clinical protocol, not an efficacy study. POINTs first proposed or significantly expanded by the authors (POINT 3: integrated lateral pterygoid/fat pad/perivascular maxillary artery/pterygopalatine region; POINT 5: intraoral medial pterygoid; POINT 7: superficial parotid fascia; POINT 9: cervical sympathetic ganglion region; POINT 10: perivascular fascia of the facial artery; POINT 11: upper posterior cervical release) substantially extend the original four-region framework. The proposed eleven-point protocol may be evaluated in future studies addressing anatomical validation, feasibility, safety, and controlled clinical effectiveness. Performance of the injection procedures is restricted to physicians with adequate training in ultrasound-guided interventions, in accordance with the legal regulations and professional scopes of practice of each country. Within this framework, dentists play a central role in the diagnosis, exclusion, and referral of non-odontogenic toothache, and physical therapists and acupuncturists collaborate in assessment and post-procedure rehabilitation. POINT 11 is an advanced, investigational technique; the present article alone must not serve as the basis for its adoption into clinical practice or education. Prospective, ideally controlled, studies will be required in the future to evaluate the clinical effectiveness of the proposed protocol.

## Figures and Tables

**Table 1:** Risk stratification and operator requirements for the eleven POINTs.

Level	POINTs	Principal at-Risk Structures	Operator Requirements	Facility Requirements
Basic	1 (masseter), 2 (temporalis), 6 (TMJ capsule complex), 7 (superficial parotid fascia)	Superficial targets at a distance from major neurovascular structures; at POINT 7, the facial nerve branches and the parotid duct lie immediately deep to the target plane, and the needle tip is kept superficial to the parotid gland capsule	Physicians with basic training in ultrasound-guided injection	Standard outpatient procedural setting
Intermediate	3 (integrated lateral pterygoid), 4 (medial pterygoid, extraoral), 5 (medial pterygoid, intraoral), 8 (digastric), 10 (perivascular fascia of the facial artery)	Maxillary artery and pterygopalatine fossa; deep intraoral structures; facial artery	Physicians with substantial experience in ultrasound-guided head and neck procedures	As above, plus emergency medications and monitoring
Advanced	9 (cervical sympathetic ganglion region), 11 (upper posterior cervical, C0–1/C1–2)	Carotid sheath and cervical sympathetic trunk; vertebral artery, PAOM, ligamentum flavum, dura mater, spinal cord	Restricted to physicians with extensive experience in head and neck interventions who have acquired supervised experience with this technique; this procedure must not be performed on the basis of this article alone	Facility with an emergency-response system (airway management, resuscitation, and transfer to a higher-level center)

**Table 2:** Contraindications, complications, and their prevention, recognition, and management.

**Part A. Contraindications**			
**Category**	**Description**		
Absolute contraindications (all POINTs)	Infection at the puncture site; severe coagulopathy; inability to obtain patient consent		
Relative contraindications (all POINTs)	Anticoagulant or antiplatelet therapy (avoided in principle in the Advanced category; an individualized risk–benefit assessment is documented); anatomical variation, including variant vascular anatomy [17]		
**Part B. Complication Prevention, Recognition, and Management**			
**Complication**	**Prevention**	**Recognition**	**Management**
Vascular puncture, bleeding, or hematoma	Color Doppler identification of vessels before each release; aspiration and hydrolocation before injection; fine-needle (27–30 G) out-of-plane technique with continuous needle-tip visualization.	Visible blood in the syringe on aspiration; expanding hypoechoic area on real-time ultrasound; skin swelling or discoloration at the puncture site; at POINTs 8 and 9, neck swelling, dysphagia, voice change, or stridor (possible airway compromise)	Immediate needle withdrawal; direct compression hemostasis (≥5 min); observation; if bleeding does not stop or the hematoma expands, transfer to a facility with emergency vascular surgery capability; if airway compromise is suspected (POINTs 8 and 9), airway assessment takes priority, with emergency airway management and immediate transfer
Vasovagal reaction	Pre-procedural screening for history of vasovagal episodes; lateral decubitus position throughout; calm procedural environment.	Pallor, diaphoresis, nausea, bradycardia, or hypotension during or immediately after the procedure	Place the patient supine with legs elevated; continuous vital-sign monitoring until recovery; intravenous access if symptoms persist
Infection	Strict aseptic technique (skin disinfection, sterile probe cover, no-touch technique); particularly rigorous for the intraoral approach (POINT 5); sterile single-use needles and syringes.	Post-procedural erythema, swelling, localized warmth, increasing pain, or fever (>38 °C) developing within days of the procedure	Blood tests (white blood cell count, C-reactive protein); ultrasound examination to rule out abscess formation; wound or aspirate culture; empirical antibiotic therapy; if an abscess is confirmed, incision and drainage with specialist (oral surgery or otolaryngology) referral; suspected deep cervical space infection warrants emergency management
Post-injection pain and delayed muscle soreness	Minimization of injection volume (1–2 mL per POINT; total ≤ approx. 8 mL); physiological saline or bicarbonate Ringer’s solution without local anesthetics.	Localized pain or soreness at the injection site, typically appearing within hours and resolving within 1–3 days	Observation and reassurance; if symptoms persist beyond 72 h, re-evaluation by ultrasound
Vertebral artery injury, dissection, or thromboembolism (POINTs 9 and 11; Advanced)	Color Doppler identification of the vertebral artery is mandatory; direct vascular puncture is strictly forbidden; the needle is not advanced unless the tip is clearly visualized; bone contact with the C1 posterior arch is avoided (a needle tip burred by bone contact can cause tissue injury); operator requires Advanced-level competency (Table 1).	Sudden severe headache, neck pain, or new neurological deficit (e.g., dizziness, visual disturbance, ataxia, contralateral weakness) during or shortly after the procedure	Immediate termination of the procedure; emergency neurological evaluation; urgent vascular imaging (CT angiography); transfer to a facility with neurovascular intervention capability
Neural injury (POINTs 9 and 11; Advanced)	Continuous needle-tip visualization; avoidance of paresthesia provocation; anatomical knowledge of the cervical sympathetic trunk, vagus nerve, and spinal nerve roots. Because no local anesthetic is used, pharmacological sympathetic blockade (e.g., Horner’s syndrome) is not expected.	New-onset numbness, paresthesia, weakness, or radicular pain during or after injection	Immediate needle withdrawal; neurological examination; if symptoms persist, specialist (neurology or neurosurgery) referral
Dural puncture with inadvertent subarachnoid injection, or epidural injection (POINT 11; Advanced)	The target is the ligamentum flavum itself (C1–C2) or the posterior atlanto-occipital membrane (C0–1); spread into the epidural space may occur but is not the intended target. Bone contact with the C1 posterior arch is avoided, because a needle tip burred by bone contact can cause tissue injury. Continuous needle-tip visualization; color Doppler identification of the vertebral artery; aspiration before injection.	Cerebrospinal fluid on aspiration; sudden onset of motor block, sensory loss below the level of injection, respiratory difficulty, or severe headache; positional headache with nausea developing within 24–48 h (post-dural puncture headache)	Immediate termination; airway management and hemodynamic support; emergency transfer. Post-dural puncture headache: bed rest, hydration, and analgesics; anesthesiology consultation (epidural blood patch) if symptoms persist
**Part C. Procedural Safeguards**			
**Item**	**Description**		
Termination criteria (all POINTs)	The needle is not advanced unless the needle tip is clearly identified (the procedure is terminated if identification cannot be regained); abnormal resistance; sudden worsening of pain or new neurological symptoms		
Post-procedural observation	30–60 min (longer for the Advanced category); at discharge, patients are instructed about neurological symptoms and bleeding and are given an emergency contact		

**Table 3:** Technical parameters of the eleven-point US-FHR protocol.

POINT	Patient Position	Probe	Approach	Needle	Volume per POINT	Typical Target Depth (from Figure)	Specific Cautions
1 Masseter	Lateral decubitus	Linear (convex advantageous for an initial overview)	Out-of-plane	27–30 G	1–2 mL	approx. 0.5–1 cm (Figure 2)	Superficial target, distant from major neurovascular structures
2 Temporalis	Lateral decubitus	Linear	Out-of-plane	27–30 G	1–2 mL	approx. 1–2 cm (Figure 3)	Muscle belly and coronoid-insertion fascia are targeted
3 Lateral pterygoid (integrated)	Lateral decubitus	Linear	Out-of-plane	30 G	1–2 mL	approx. 2.5–3 cm (Figure 4)	Color Doppler identification of the maxillary artery is mandatory
4 Medial pterygoid (extraoral)	Lateral decubitus, affected side up; wide mouth opening	Convex (panoramic view)	Out-of-plane	27 G, 38 mm	1–2 mL	approx. 2.5–3 cm (Figure 5b)	Needle inserted anterior to the coronoid process; deep course medial to the mandibular ramus
5 Medial pterygoid (intraoral)	Lateral decubitus	Convex (C5-2; Figure 6)	Intraoral route	30 G	0.5–1 mL	approx. 3 cm (Figure 6)	Strict intraoral aseptic technique; collaboration with a dentist is essential
6 TMJ capsule complex	Lateral decubitus	Linear	Out-of-plane	27–30 G	1–2 mL	approx. 0.5–1 cm (Figure 7)	Condylar process, articular disk, and joint capsule identified; joint dynamics confirmed during opening and closing
7 Superficial parotid fascia	Lateral decubitus	Linear	Out-of-plane	30 G	0.5–1 mL	approx. 0.5 cm (Figure 8)	The facial artery and posterior auricular artery must be avoided; needle tip kept superficial to the parotid capsule
8 Digastric	Lateral decubitus	Linear	Out-of-plane	27–30 G	1–2 mL	approx. 0.5–1 cm (Figure 9)	Hyoid bone and submandibular gland serve as landmarks
9 Cervical sympathetic ganglion region	Lateral decubitus	Linear	Out-of-plane	27–30 G	1–2 mL	approx. 1.5–2 cm (Figure 10)	Internal jugular vein and common carotid artery confirmed; Advanced (Table 1)
10 Facial artery	Lateral decubitus	Linear	Out-of-plane	30 G	0.5–1 mL	approx. 1 cm (Figure 11)	Color Doppler identification of the facial artery is mandatory; injection lateral to the arterial sheath
11 Upper posterior cervical	Lateral decubitus	Convex (C5-2)	Out-of-plane (posterior)	30 G	1–2 mL	approx. 2.5–3 cm (Figure 12, Figure 13 and Figure 14)	Color Doppler identification of the vertebral artery is mandatory; direct vascular puncture strictly forbidden; Advanced (Table 1)

**Table 4:** Supporting evidence for each of the eleven POINTs.

POINT	Supporting Evidence
1 Masseter	Published referred-pain pattern to the teeth [7,9,10] + expert clinical experience
2 Temporalis	Published referred-pain pattern [7,9] + expert clinical experience
3 Lateral pterygoid/fat pad/maxillary artery/pterygopalatine (integrated)	Published referred-pain pattern for the lateral pterygoid [7]; the integrated target is proposed in this protocol
4 Medial pterygoid (extraoral)	Published referred-pain pattern [7] + expert clinical experience
5 Medial pterygoid (intraoral)	Newly proposed approach in this protocol; referred-pain pattern as for POINT 4 [7]
6 TMJ capsule complex	Referred pain documented in the TMD literature [9,14] + expert clinical experience
7 Superficial parotid fascia	Author-proposed target (hypothesis-generating) [3]
8 Digastric muscle	Published referred-pain pattern [7] and a case report of anterior-belly referral to the mandibular anterior teeth [18] + expert clinical experience
9 Cervical sympathetic ganglion region	Hypothesis-generating; application to non-odontogenic toothache proposed by the authors; Advanced (Table 1)
10 Perivascular fascia of the facial artery	Author-proposed target (hypothesis-generating)
11 Upper posterior cervical (C0–1/C1–2)	Hypothesis-generating; extrapolated from lumbar ligamentum flavum findings [19], as explicitly stated; Advanced (Table 1)

## Data Availability

No new datasets were generated or analyzed in this study; it is a clinical protocol and narrative synthesis. The demonstration videos (Videos S1 to S11) are openly available at Zenodo (DOI: 10.5281/zenodo.21318086; https://doi.org/10.5281/zenodo.21318086).

## Supplementary Materials

Demonstration videos of the US-FHR procedures at each POINT (Videos S1 to S11) are openly available at Zenodo (DOI: 10.5281/zenodo.21318086; https://doi.org/10.5281/zenodo.21318086). POINT 11 is presented as two videos (S11A and S11B), reflecting the selection between C0–1 (vertebral artery curvature) and C1–C2 (ligamentum flavum) based on tender-point dominance, both forming a single conceptual procedure. Video S1: POINT 1—Masseter (superficial and deep layers) US-FHR. Video S2: POINT 2—Temporalis US-FHR. Video S3: POINT 3—Lateral pterygoid/fat pad/maxillary artery/pterygopalatine integrated US-FHR. Video S4: POINT 4—Medial pterygoid (extraoral approach) US-FHR. Video S5: POINT 5—Medial pterygoid (intraoral approach) US-FHR. Video S6: POINT 6—Temporomandibular joint capsule complex US-FHR. Video S7: POINT 7—Superficial parotid fascia US-FHR. Video S8: POINT 8—Digastric muscle US-FHR. Video S9: POINT 9—Cervical sympathetic ganglion region (centered on the middle cervical ganglion, extending cephalad and caudad to include the superior cervical and stellate ganglia) US-FHR. Video S10: POINT 10—Perivascular fascia of the facial artery US-FHR. Video S11A: POINT 11—Upper posterior cervical release (C0–1 vertebral artery curvature, tender-side example) US-FHR. Video S11B: POINT 11—Upper posterior cervical release (C1–C2 ligamentum flavum/dural region, tender-side example) US-FHR.

## Abbreviations

The following abbreviations are used in this manuscript:

APF	aponeurotic fascia
CDS	Crowned Dens Syndrome
DC/TMD	Diagnostic Criteria for Temporomandibular Disorders
EPI	epimysium FHR fascia hydrorelease
FPS	Fascial Pain Syndrome
ICOP	International Classification of Orofacial Pain
MPS	myofascial pain syndrome
NDT	non-odontogenic toothache
PAOM	posterior atlanto-occipital membrane
SMAS	superficial musculoaponeurotic system
TGN	trigeminal neuralgia
TMD	temporomandibular disorder
TPI	trigger-point injection
TrP	trigger point
US-FHR	ultrasound-guided fascia hydrorelease
